# Rewired type I IFN signaling is linked to age-dependent differences in COVID-19

**DOI:** 10.1016/j.xcrm.2025.102285

**Published:** 2025-08-19

**Authors:** Lev Petrov, Sophia Brumhard, Sebastian Wisniewski, Philipp Georg, David Hillus, Anna Hiller, Rosario Astaburuaga-García, Nils Blüthgen, Emanuel Wyler, Katrin Vogt, Hannah-Philine Dey, Saskia von Stillfried, Christina Iwert, Roman D. Bülow, Bruno Märkl, Lukas Maas, Christine Langner, Tim Meyer, Jennifer Loske, Roland Eils, Irina Lehmann, Benjamin Ondruschka, Markus Ralser, Jakob Trimpert, Peter Boor, Sammy Bedoui, Christian Meisel, Marcus A. Mall, Victor M. Corman, Leif Erik Sander, Jobst Röhmel, Birgit Sawitzki

**Affiliations:** 1 Berlin Institute of Health (BIH) at Charité, Charité – Universitätsmedizin Berlin, Berlin, Germany; 2 Department of Infectious Diseases, Respiratory Medicine and Critical Care, Charité – Universitätsmedizin Berlin, Berlin, Germany; 3 Department of Pediatric Respiratory Medicine, Immunology and Critical Care Medicine, Charité – Universitätsmedizin Berlin, Berlin, Germany; 4 Institute of Pathology, Charité – Universitätsmedizin Berlin, Berlin, Germany; 5 IRI Life Sciences, Humboldt-Universität zu Berlin, Berlin, Germany; 6 Max-Delbrück-Center for Molecular Medicine in the Helmholtz Association, Berlin Institute for Medical Systems Biology, Berlin, Germany; 7 Institute of Pathology, RWTH Aachen University Hospital, Aachen, Germany; 8 Institute of Pathology, Medical Faculty Augsburg, Augsburg University, Augsburg, Germany; 9 Institute of Virology, Freie Universität Berlin, Berlin, Germany; 10 Department of Nephrology and Immunology, RWTH Aachen University Hospital, Aachen, Germany; 11 Department of Immunology, Labor Berlin, Charité Vivantes, Berlin, Germany; 12 Center for Digital Health, Berlin Institute of Health (BIH), Charité – Universitätsmedizin Berlin, Berlin, Germany; 13 German Center for Lung Research (DZL), Associated Partner Site Berlin, Berlin, Germany; 14 Institute of Legal Medicine, University Medical Center Hamburg- Eppendorf, Hamburg, Germany; 15 Department of Biochemistry, Charité – Universitätsmedizin Berlin, Berlin, Germany; 16 Wellcome Centre for Human Genetics, University of Oxford, Oxford, UK; 17 Department of Microbiology and Immunology at the Doherty Institute for Infection and Immunity, The University of Melbourne, Melbourne, VIC, Australia; 18 Institute of Experimental Immunology, University of Bonn, Berlin, Germany; 19 German Center for Child and Adolescent Health, Partner Site Berlin, Berlin, Germany; 20 Institute of Virology, Charité-–Universitätsmedizin Berlin, Corporate Member of Freie Universität Berlin, Humboldt-Universität zu Berlin, and Berlin Institute of Health, Berlin, Germany; 21 German Centre for Infection Research (DZIF), Partner Site Charité, Berlin, Germany

**Keywords:** type I IFN, signaling, age, children, COVID-19, SARS-CoV-2, STAT1, STAT3, monocytes, T cells, B cells, antibodies, immune response

## Abstract

Advanced age is the most important risk factor for severe disease or death from COVID-19, but a thorough mechanistic understanding of the molecular and cellular underpinnings is lacking. Multi-omics analysis of 164 samples from SARS-CoV-2-infected persons aged 1 to 84 years reveals a rewiring of type I interferon (IFN) signaling with a gradual shift from signal transducer and activator of transcription 1 (STAT1) to STAT3 activation in monocytes, CD4^+^ T cells, and B cells with increasing age. Diversion of IFN signaling is associated with increased expression of inflammatory markers, enhanced release of inflammatory cytokines, and delayed contraction of infection-induced CD4^+^ T cells. A shift from IFN-responsive germinal center B (GCB) cells toward CD69^high^ GCB and atypical B cells during aging correlates with immunoglobulin (Ig)A production in children, whereas complement-fixing IgG predominates in adults. Our data provide a mechanistic basis for inflammation-prone responses to infections and associated pathology during aging.

## Highlights

• Aging shifts IFN signaling from STAT1 to inflammatory STAT3 activation
• Children’s cells show stronger ISG profiles and exclusively follicular responses
• Adults exhibit additional extrafollicular B cell activation and high cytokine levels
• IFN signaling rewiring explains age-linked COVID-19 immune pathology

## Graphical abstract

**Figure:**
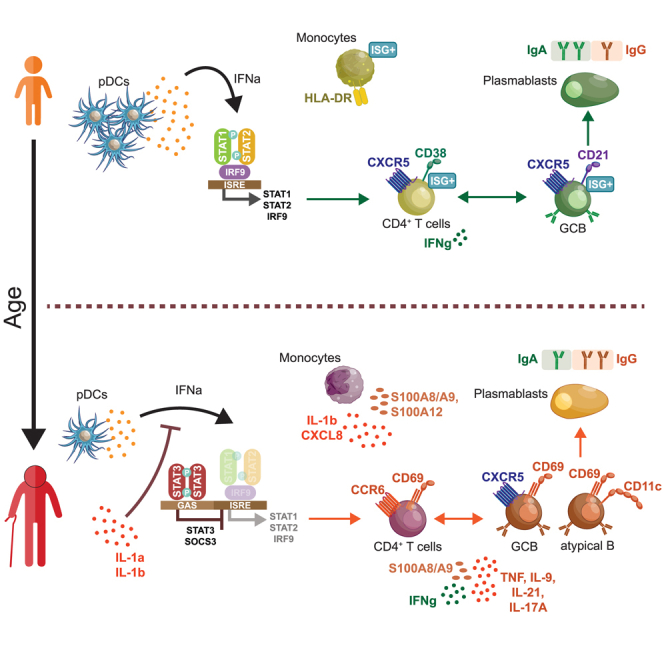

## Teaser

Petrov et al. identify an age-dependent shift in interferon signaling during SARS-CoV-2 infection, with older individuals favoring a STAT3-driven inflammatory response. This mechanistic insight helps explain why COVID-19 leads to more severe outcomes in adults than in children.

## Introduction

Age is a known modulator of immune responses, highlighted during the COVID-19 pandemic when older adults faced higher risk of severe disease, while young children experienced mostly mild or asymptomatic courses.^1^^,^^2^^,^^3^^,^^4^^,^^5^^,^^6^^,^^7^

Our earlier work showed stronger type I interferon (IFN) and interferon-stimulated gene (ISG) signatures in epithelial and innate immune cells of SARS-CoV-2-infected children, suggesting enhanced early viral control.^8^ Yet, viral loads and clearance kinetics appeared similar across ages.^8^^,^^9^

Adaptive immunity is essential for long-term SARS-CoV-2 control.^10^ T cells aid clearance, and neutralizing antibodies reduce reinfection risk.^11^ Most children seroconvert and develop specific T cells without systemic inflammation, except in rare multisystem inflammatory syndrome in children cases.^10^^,^^12^^,^^13^^,^^14^^,^^15^^,^^16^ In contrast, severe COVID-19 in older adults involves immune dysregulation, including myeloid cell dysfunction and hyperactive cytotoxic T cells, suggesting qualitative differences in immune cell activation with age.^17^^,^^18^ However, the molecular basis of these differences remains unclear.

The pandemic created a unique opportunity to investigate age-dependent immune responses to a novel virus without the confounding effects of pre-existing immunity.^5^^,^^19^^,^^20^ To explore this, we conducted a multi-omics analysis of children and adults infected early in the pandemic. The RECAST study (resilience of children compared to adults in SARS-CoV-2 infection) enabled direct comparison of immune responses within households.^5^ We also performed *in vitro* assays using cells from uninfected donors.

We found a continuous age-related shift in type I IFN signaling from STAT1- to STAT3-driven pathways in monocytes, T, and B cells. This rewiring correlated with changes in inflammatory gene expression across immune cells, promoting systemic inflammation in older individuals. Features included expansion of S100A8/A9^+^ and CXCL8^+^ HLA-DR^low^ monocytes, increased CCR6^+^ peripheral T helper cells, and CD69^+^ germinal center and atypical B cells. In children, responses were more targeted and follicular in nature, marked by CD38^high^CXCR5^high^ T follicular helper cells, germinal center B cells, and rapid T cell contraction.

Our study offers a comprehensive age-stratified profile of SARS-CoV-2 immune responses, revealing a progressive shift from antiviral STAT1 to proinflammatory STAT3 signaling with age. This mechanistic insight helps explain the heightened susceptibility of older adults to severe viral disease.

## Results

### Upregulation of HLA-DR versus CCR6 distinguishes infection-induced monocytes in children and adults

**Figure 1:**
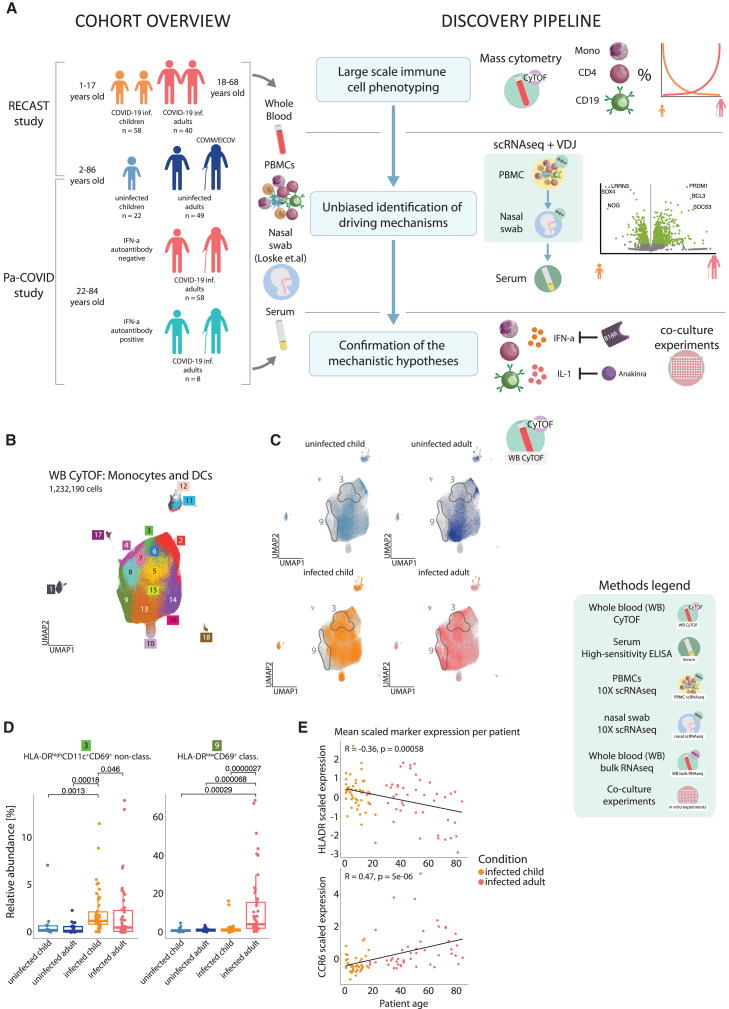
Distinct age-dependent patterns of monocyte activation are characterized by opposite HLA-DR and CD11c versus CCR6 expression (A) Overview of the study cohort and methodological pipeline. Blood samples were collected from SARS-CoV-2-infected children and adults as well as controls spanning an age range from 1 to 86 years. Whole-blood mass cytometry (CyTOF) and scRNA-seq combined with VDJ (Variable, Diversity, and Joining)-seq-based clonotype identification were used to determine age-specific alterations in the monocyte, T cell, and B cell compartment. The obtained results together with serum antibody profiles were used to develop hypotheses on their functional properties, inducing mechanisms and transcriptional control, which were tested in *ex vivo* cultures. Detailed information on samples included in all reported assays can be found in Table S1. Additional cohort summary is included in Table S2. Multiple icons are used throughout the paper to identify data from different experiments (Methods legend in the lower right corner of the figure). (B) UMAP (Uniform Manifold Approximation and Projection), showing pre-gated monocyte and dendritic cell (DC) populations from the CyTOF dataset. 18 clusters have been produced using a semi-supervised approach with FlowSOM algorithm. (C) UMAPs, showing the location of cells belonging to the respective group (colored in, whereas gray identifies cells in other groups). Gray outlines indicate cluster regions enriched in infected children or adults. (D) Boxplots, showing relative abundance of infection-induced clusters resulting from the FlowSOM algorithm, calculated per sample within all monocytes and DCs from the CyTOF data. Kruskal-Wallis + Wilcoxon *p* values. (E) Scatterplots, showing mean *Z* score (calculated by subtracting population mean from value divided by standard deviation) normalized HLA-DR and CCR6 expression in relationship to patient age for infected patients (linear model fitted to data and Spearman’s rank correlation coefficient in black) within monocyte and DC CyTOF data.

To study the age-dependent differences in antiviral immune cell responses, we established a unique cohort early in the pandemic consisting of SARS-CoV-2-infected children and adults (Figure 1A). This cohort includes patients who were enrolled in three observational studies. RECAST allowed us to perform a multi-omics-based profiling of specimens collected from infected and non-infected control families spanning an age range from 1 to 68 years.^5^ None of the infected patients experienced severe disease. Most of the studied infected individuals (children and adults, 106 out of the 164) were enrolled into RECAST. However, the age range of this household study was limited (1–68 years) and did not allow us to measure samples from patients older than 68 years. To extend the age range up to 84 years of age, and thus capture the age group most severely affected by COVID-19, we included additional 58 patients from the previously described PA-COVID study, which enrolled SARS-CoV-2-infected patients who were hospitalized either for isolation or treatment of the infection-related symptoms.^19^ Analysis of specimens collected from the prospective vaccination trial EICOV/COVIMMUNIZE studies enabled integration of data from older non-infected controls (up to 86 years).^19^^,^^20^ Control patients were tested negative for SARS-CoV-2 and had no clinical signs of an infection.

We focused on samples collected within 2 weeks of symptom onset, or infection in asymptomatic children, to identify immune cell patterns linked to early age-related symptom severity (mean days post-symptom onset, DPSO: children 5 ± 3.3, adults 7.7 ± 4.5). COVID-19 severity was defined according to the World Health Organization (WHO) ordinal scale.^17^^,^^18^^,^^21^ Tables S1 and S2 provide patient-level data, including DPSO, comorbidities, and assay group compositions.

Given our prior finding that dysregulated myeloid cells are a hallmark of severe COVID-19, we examined monocyte and dendritic cell (DC) responses using mass cytometry (CyTOF) on whole blood (Figures 1B–1E and S1A–S1C).^17^ FlowSOM clustering identified plasmacytoid (CD14^−^CD123^+^CD11c^−^) and conventional DCs (CD14^−^CD123^−^CD11c^+^); classical, intermediate, and non-classical monocytes; and Ki67^+^ hematopoietic stem and progenitor cells (HSPCs) from pre-gated CD45^+^CD15^−^CD3^−^CD56^−^CD19^−^ cells. SARS-CoV-2 infection shifted monocyte/DC cluster composition by age: infected children had more plasmacytoid dendritic cells (pDCs) and classical dendritic cells (cDCs) (Figure S1B) and higher proportions of HLA-DR^high^CD11c^+^CD69^+^ non-classical monocytes (cluster 3; Figures 1D, S1A, and S1B). In contrast, infected adults had more HLA-DR^low^ classical monocytes expressing CD10, CD69, and CCR6 (cluster 9).

Overall, monocyte and DC profiles differed markedly between age groups. Infected children showed higher expression of HLA-DR, CD11c, and CD123, while adults had elevated CD69, CD226, CD10, CXCR3, and CCR6 (Figure S1C). HLA-DR levels across monocytes declined with age, especially after 60 (Figure 1E, upper), while CCR6 expression increased with age (Figure 1E, lower). These findings indicate that monocyte responses to SARS-CoV-2 are age dependent.

### Gradual age-dependent changes in the phenotype of infection-induced CD4^+^ T and B cells

**Figure 2:**
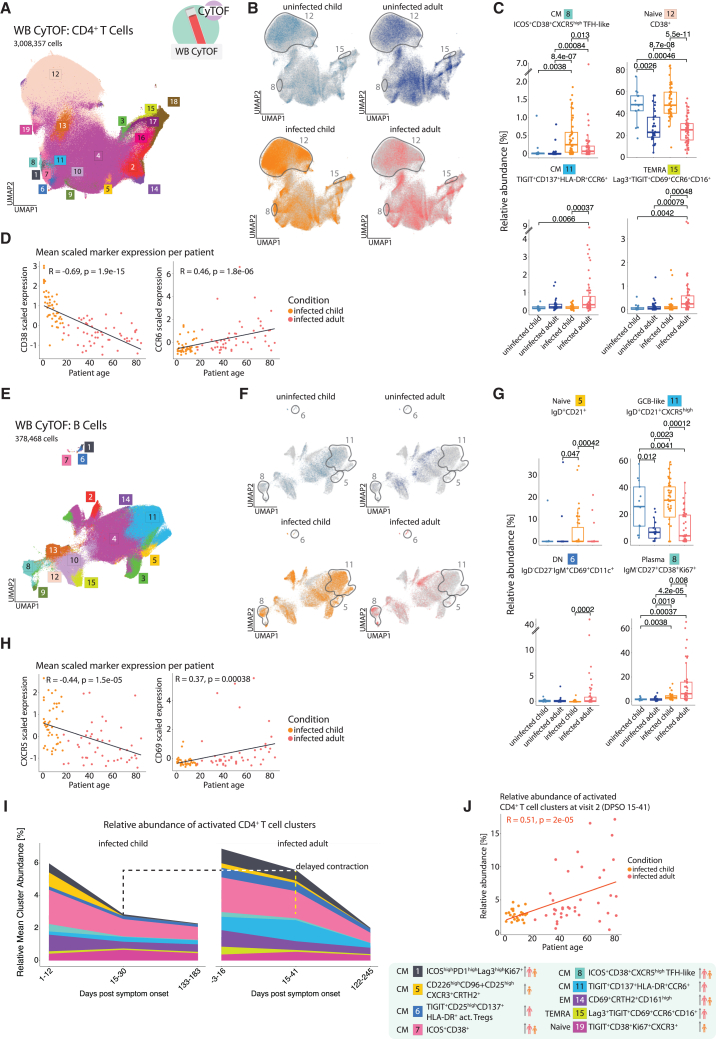
Gradual age-dependent change in the phenotype of infection-induced CD4^+^ T cells and B cells (A) UMAP, showing pre-gated CD4^+^ T cells from the CyTOF dataset. 19 clusters have been produced using a semi-supervised approach with FlowSOM algorithm. UMAP presents all the patients that were part of the dataset and used for clustering, including follow-up measurements of some patients done approximately 2 weeks and 6 months after the first, acute infection phase measurement (details in Table S1). (B) UMAPs, showing the location of cells belonging to the respective group (colored in, whereas gray identifies cells in other groups). Gray outlines indicate cluster regions enriched in infected children or adults. (C) Boxplots, showing relative abundance of infection-induced clusters resulting from the FlowSOM algorithm, calculated per sample within all CD4^+^ T cells from the CyTOF data. Kruskal-Wallis + Wilcoxon *p* value. (D) Scatterplots, showing mean *Z* score normalized CD38 and CCR6 expression in relationship to patient age for infected patients (linear model fitted to data and Spearman’s rank correlation coefficient in black) within CD4^+^ T cell CyTOF data. (E) UMAP, showing pre-gated B cells from the CyTOF dataset. 15 clusters have been produced using a semi-supervised approach with FlowSOM algorithm. (F) UMAPs, showing the location of cells belonging to the respective group (colored in, whereas gray identifies cells in other groups). Gray outlines indicate cluster regions enriched in infected children or adults. (G) Boxplots, showing relative abundance of infection-induced clusters resulting from the FlowSOM algorithm, calculated per sample within all B cells from the CyTOF data. Kruskal-Wallis + Wilcoxon *p* values. (H) Scatterplots, showing mean *Z* score normalized CXCR5 and CD69 expression in relationship to patient age for infected patients (linear model fitted to data and Spearman’s rank correlation coefficient in black) within B cell CyTOF data. (I) Time-dependent stacked line graphs, displaying the relative mean cluster abundance of all activated CD4^+^ T cell clusters determined by CyTOF. Activated clusters were defined as clusters having above-average *Z*-scored expression of activation markers (CD25, HLA-DR, CD38, CD137, CD69, and Ki67) compared to other clusters. Patient group color-coded figurines on the right-hand side point out cluster accumulation patterns. (J) Scatterplot, showing the sum of the relative abundance of all activated CD4^+^ T cell clusters determined by CyTOF.

To assess whether monocyte responses were linked to T cell differences in SARS-CoV-2-infected children and adults, we performed CD4^+^ T cell subclustering using CyTOF data (Figures 2A and S2A), identifying 19 clusters grouped into naive, central memory (CM), effector memory (EM), and terminally differentiated effector memory (TEMRA) subsets based on CD62L and CD45RO expression. Among them, peripheral helper clusters with high CCR6, CD69, and CD16 expression (clusters 11 and 15) were enriched in infected adults in accordance to our previous findings (Figures 2B, 2C, and S2B).^18^ Infected children showed higher frequencies of CD38^high^CXCR5^high^ follicular helper T cells (cluster 8) and CD38^+^ naive CD4^+^ T cells (cluster 12). Accordingly, CD38 expression was elevated in children, while CCR6 was higher in adults (Figure S2C). These markers showed opposing, linear age correlations, with crossover at ∼40 years (Figure 2D). This divergence was also observed between children and their own parents (Figure S2D), arguing against shared genetic or environmental causes.

We next analyzed B cell phenotypes (Figures 2E and S3A), identifying 15 clusters classified into transitional, naive, GCB-like, double-negative (DN), and memory B cells as well as plasmablasts based on immunoglobulin (Ig)D, IgM, CD27, CD10, CD38, and CXCR5 expression. Infected adults had increased CD69-expressing DN-like B cells (clusters 1 and 6) and CD69^+^ GCB-like naive B cells (cluster 14) (Figures 2F, 2G, and S3B).^22^ Clusters 1 and 6 were CD11c^+^DN2-like atypical B cells, while cluster 14 contained low IgD- and CD21-expressing follicular B cells.^22^^,^^23^

Thus, both follicular and extrafollicular B cell responses were promoted in infected adults, linked with strong upregulation of CD69. These changes were associated with more IgM^−^ plasmablasts (cluster 8).

In contrast, infected children had more transitional (cluster 3), naive (cluster 5), and GCB-like naive B cells (cluster 11), indicating exclusive follicular responses (Figures 2G, S3B, and S3D). IgD expression was higher in B cells from children (Figure S3D). Similar to T cells, these patterns were mirrored between children and their parents (Figure S3C), with CXCR5 and CD21 decreasing and CD69 increasing with age (Figure 2H; Figure S3E).

To rule out sampling time as a confounder, we examined the impact of DPSO. Eleven of 58 infected children were asymptomatic, making DPSO unavailable. For symptomatic cases, CyTOF clusters showed no consistent DPSO correlation (Mendeley Figure 1). Later sampling time points did not reverse age-specific CD4^+^ T cell phenotypes (Figures 2I and 2J). For instance, child-specific clusters (e.g., 5 and 8) remained absent in adults, and adult-specific clusters (e.g., 11 and 15) stayed low in children. While initial CD4^+^ T cell activation was comparable, children exhibited faster contraction (Figure 2J).

Finally, averaged marker intensities across monocytes, CD4^+^ T cells, and B cells confirmed age-related activation shifts (Figure S3F). CD38 and CXCR5 were higher in children’s lymphocytes, whereas CD69 and CCR6 were higher in adult lymphocytes and monocytes, supporting a system-wide age-associated immune response pattern.

### Age-dependent shift from type I IFN responsive to inflammatory monocytes

**Figure 3:**
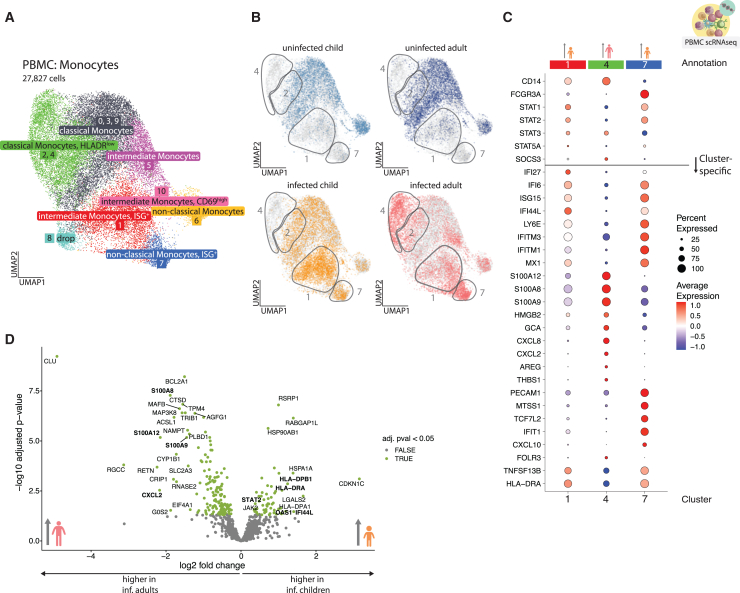
Age-dependent shift from type I IFN responsiveness to increased inflammatory potential (A) UMAP, showing monocyte cells, subset from the PBMC scRNA-seq data. 11 clusters have been produced using a graph-based approach as implemented in Seurat package (k-nearest-neighbors [KNN] graph with Louvain community detection). Some clusters share annotation due to phenotypical similarity and accumulation pattern and are treated as one population for abundance testing in S3E. Clusters annotated as “dropped” were of low quality (as concluded from inspecting number of features, no. of counts, and percentage of mitochondrial genes as well as other population-specific genes). (B) UMAPs, showing the location of cells belonging to the respective group (colored in, whereas gray identifies cells in other groups). Gray outlines indicate cluster regions enriched in infected children or adults. (C) Dotplot, showing scaled average expression of genes in monocyte clusters, increased with infection (1, 4, and 7) in cells belonging to infected patients. A horizontal line splits the dotplot in two parts; genes above the line were curated based on the presence of clusters with pronounced ISG signature and include other genes useful for annotation; genes below the line were found to be differentially expressed between the clusters (FindMarkers Seurat function). (D) Volcano plot, showing the results of differential expression (DE) analysis of monocytes from clusters expanded with infection (1, 4, and 7) using DESeq2. Significantly enriched genes (baseMean counts over 50 and adjusted *p* value < 0.05, a total of 189 genes) are colored green, and 20 significant genes with the highest absolute log2 fold change are labeled. Additional significant genes, tangential to other findings, were labeled *ad hoc*.

Upregulation of HLA-DR expression (Figures 1; S1) can be induced by type I and II IFN signaling.^24^ To investigate potential differences in IFN responsiveness between cells from children and adults, we performed single-cell RNA sequencing (scRNA-seq) of peripheral blood mononuclear cells (PBMC) samples (Figures S4A–S4C) with subsequent subclustering of the monocytes (Figures 3A and S4D). We identified 11 monocyte clusters. The cell clusters were initially assigned to known differentiation states based on the established gene markers *CD14* and *FCGR3A* into classical (*CD14*^*+*^*FCGR3A*^*−*^), intermediate (*CD14*^*+*^*FCGR3A*^*+*^), and non-classical (*CD14*^*low*^*FCGR3A*^*+*^) monocytes. We used top 10 differentially expressed markers in each cluster, like *HLADR*, to further annotate the populations (Figure 3C, only infection-induced clusters shown, complete cluster-specific marker list in Table S4).

HLA-DR^low^ classical monocytes were increased in infected adults (clusters 2 and 4), whereas ISG^+^ intermediate (cluster 1) and non-classical (cluster 7) monocytes were observed almost exclusively in children (Figures 3A, 3B, S4D, and S4E). Genes characterizing those clusters included *IFI27*, *IFI6*, and *ISG15* but also *SIGLEC1*, which had been previously linked with mild COVID-19.^25^ Importantly, type I IFN serum concentrations were indistinguishable between children and adults, indicating a difference in IFN responsiveness, not production. (Figure S4G).

Notably, ISG^+^ non-classical monocytes were expanded primarily in asymptomatic children, suggesting enhanced IFN responsiveness may reduce disease severity (Mendeley Figure 2). HLA-DR^low^ monocyte clusters observed in adults aligned with our CyTOF findings (Figures 1D and 1E). Cluster 4 also showed high transcription of proinflammatory mediators S100A8/9/12 and chemokines CXCL8 and CXCL2 (Figures 3C and 3D).

Gene set enrichment analysis (GSEA) of adult monocytes revealed enrichment of “neutrophil-mediated immunity” (Figure S4F), consistent with elevated serum CXCL8, interleukin (IL)-1b, IL-6, and tumor necrosis factor (TNF) in adults versus children (Figure S4G). In contrast, children’s ISG^+^ monocytes showed high *STAT1*/*STAT2* expression, and *STAT2* was broadly elevated across all monocytes in infected children (Figure 3D), confirming increased IFN responsiveness.

### Molecular characterization of SARS-CoV-2 infection-induced T cells reveals age-dependent differences in STAT involvement and differentiation

**Figure 4:**
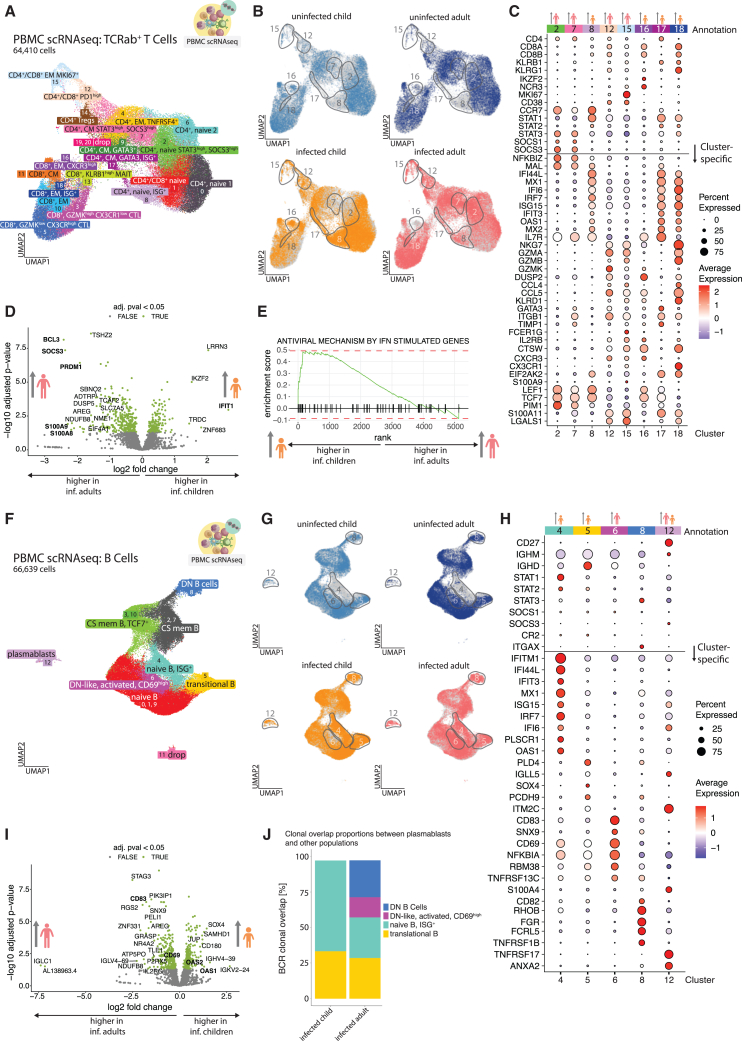
Molecular characterization of infection-associated TCRab^+^ T cells and B cells indicates increased STAT3 involvement and altered differentiation during aging (A) UMAP, showing TCRab^+^ T cells, subset from the PBMC scRNA-seq data. 21 clusters have been produced using a graph-based approach as implemented in Seurat package (KNN graph with Louvain community detection). Clusters annotated as “dropped” were of low quality (as concluded from inspecting number of features, no. of counts, and percentage of mitochondrial genes as well as other population-specific genes) (details in Table S1). (B) UMAPs, showing the location of cells belonging to the respective group (colored in, whereas gray identifies cells in other groups). Gray outlines indicate cluster regions enriched in infected children or adults. (C) Dotplot, showing scaled average expression of genes in TCRab^+^ T cell clusters, increased with infection (2, 7, 8, 12, 15, 16, 17, and 18) for cells of infected patients. A horizontal line splits the dotplot in two parts; genes above the line were curated based on the presence of clusters with pronounced ISG signature and include other genes useful for annotation; genes below the line were found to be differentially expressed between the clusters (FindMarkers Seurat function). (D) Volcano plot, showing the results of differential expression (DE) analysis of TCRab^+^ T cells from clusters expanded with infection (2, 7, 8, 12, 15, 16, 17, and 18) using DESeq2. Significantly enriched genes (baseMean counts over 50 and adjusted *p* value < 0.05, a total of 464 genes) are colored green, and 20 significant genes with the highest absolute log2 fold change are labeled. Additional significant genes, tangential to other findings, were labeled *ad hoc*. (E) Gene set enrichment analysis (GSEA) applied to TCRab^+^ T cells from the PBMC scRNA-seq experiment and clusters, expanded with infection (2, 7, 8, 12, 15, 16, 17, and 18). GSEA was done using genes from the infected adults—infected children comparison with baseMean counts > 50 (total of 5,181 genes). R package fgsea was used with Reactome gene list R-HSA-1169410 (Table S4). Genes are shown as ticks on a bold black line and ranked by log2 fold change. (F) UMAP, showing B cells, subset from the PBMC scRNA-seq data. 13 clusters have been produced using a graph-based approach as implemented in Seurat package (KNN graph with Louvain community detection). Some clusters share annotation due to phenotypical similarity and accumulation pattern and are treated as one population for abundance testing in S4E. Clusters annotated as “dropped” were of low quality (as concluded from inspecting number of features, no. of counts, and percentage of mitochondrial genes as well as other population-specific genes). (G) UMAPs, showing the location of cells belonging to the respective group (colored in, whereas gray identifies cells in other groups). Gray outlines indicate cluster regions enriched in infected children or adults. (H) Dotplot, showing scaled average expression of genes in B cell clusters, increased with infection (4, 5, 6, 8, and 12) for cells of infected patients. A horizontal line splits the dotplot in two parts; genes above the line were curated based on the presence of clusters with pronounced ISG signature and include other genes useful for annotation; genes below the line were found to be differentially expressed between the clusters (FindMarkers Seurat function). (I) Volcano plot, showing the results of differential expression (DE) analysis of B cells from clusters expanded with infection (4, 5, 6, 8, and 12) using DESeq2. Significantly enriched genes (baseMean counts over 50 and adjusted *p* value < 0.05, a total of 482 genes) are colored green, and 20 significant genes with highest absolute log2 fold change are labeled. Additional significant genes, tangential to other findings, were labeled *ad hoc*. (J) Stacked bar chart, showing proportions of cluster labels, given to the cells with callable B cell receptor (BCR) clonal identity that overlaps with at least one of the cells in the plasmablast population (cluster 12), calculated over all expanded B cell clusters.

We next asked whether altered type I IFN responsiveness could also account for the age-dependent T cell phenotypes and subclustered T cell receptor (TCR)αβ^+^ T cells from the PBMC scRNA-seq data (Figures 4A and S5A).

Clusters were first classified by coreceptor expression as CD4^+^, CD8^+^, or CD4^+^/CD8^+^ (Figure S5). Regulatory T cells (*FOXP3*^*+*^*IL-2RA*^*high*^) and mucosal-associated invariant T cells (*KLRB1*^*high*^*GZMK*^*high*^*CCR6*^*+*^*RORC*^*+*^*)* were identified based on transcript profiles. Remaining T cells were grouped into naive (*SELL*^*high*^*CCR7*^*high*^*HOPX*^*−*^), CM (*SELL*^*high*^*CCR7*^*low*^*HOPX*^*−*^), EM (*SELL*^*low*^*CCR7*^*low*^*HOPX*^*+*^), and cytotoxic lymphocytes (CTLs, *CD8A*^*+*^*CD8B*^*+*^*GZMA*^*high*^*GZMB*^*high*^*PRF1*^*high*^), using standard markers (Figure S5A).

Finally, top 10 differentially expressed genes per cluster were used to refine annotations based on mRNA profiles, such as ISG expression or high STAT3 and SOCS3 levels (Figure 4C; Table S4). Among 21 T cell clusters, ISG^+^ CD4^+^ and CD8^+^ clusters (8, 17, and 18) were enriched in children, while *STAT3*^*high*^*SOCS3*^*high*^, *PD1*^*high*^, and proliferative clusters (2, 7, 12, and 15) were predominant in adults (Figures 4B, 4C, and S5B).

Differential gene expression showed elevated *PRDM1*, *BCL3*, and *SOCS3* in adult T cells (Figure 4D). *PRDM1* (BLIMP-1) inhibits follicular helper T cell differentiation, supporting CyTOF findings of reduced CXCR5 and increased CCR6 in adults.^26^^,^^27^^,^^28^ *SOCS3*, a STAT3-inducible inhibitor of JAK-STAT signaling, limits *STAT1* activation.^29^^,^^30^^,^^31^ STAT3 further amplifies its own transcription, forming a positive feedback loop.^29^^,^^32^ In contrast, canonical type I IFN signaling via the ISGF3 complex induces STAT1, STAT2, and IRF9 expression.^30^ Accordingly, child-enriched ISG^+^ clusters showed high *STAT1/2* expression, while adult-enriched clusters showed elevated *STAT3*, *SOCS*1, and *SOCS3* (Figure 4C).

*BCL3*, a known *STAT3* activator, was also increased in adults and linked to phosphorylated STAT3 (Figure 4D).^33^^,^^34^ As in monocytes, T cells from adults showed upregulation of proinflammatory genes *S100A8* and *S100A9*. GSEA confirmed the selective enrichment of ISG^+^ T cells in infected children (Figure 4E).

Comparing infection-induced TCRab^+^ T cells, adults exhibited higher average *STAT3*, *SOCS1*, and *SOCS3* expression, whereas *STAT1* and *STAT2* were elevated in cells from infected children (Figure S5C), highlighting divergent IFN signaling with age.

### Divergent molecular profiles of infection-induced B cells translate into different plasmablast differentiation programs

To understand whether the disparate B cell phenotypes could also be aligned to differences in type I IFN responsiveness, we analyzed gene transcription in CD19^+^ B cells in our scRNA-seq data (Figures 4F–4J, S5D, and S5E). We identified 13 B cell clusters, which we merged into 8, based on transcriptional similarity (Figures 4F–4H, S5D, and S5E). We first assigned the clusters to B cell differentiation states using established markers like transitional (*IGHD*^*+*^*IGHM*^*+*^*CD38*^*+*^*CD24*^*+*^), naive (*IGHD*^*+*^*IGHM*^*+*^*CD38*^*−*^), class-switched memory ( *IGHD*^*-*^*IGHG*^*+*^*IGHA*^*+*^*CD27*^*high*^), and plasmablasts (*CD19*^*low*^*MS4A1*^*-*^*CD38*^*high*^*IGHG*^*+*^*IGHA*^*+*^) (Figure S5D). Top 10 differentially expressed markers in each cluster, like *CD69*, were used to further subdivide them (Figures 4H; Table S4). Atypical B cells lacking *CR2* (CD21) mRNA were annotated as DN (*IGHD*^*-*^*CD27*^*−*^) or DN-like activated (*CD27*^*−*^*CD69*^*high*^) cells.

Consistent with CyTOF data, we observed increased *CD69*^*+*^ B cells in infected adults (cluster 6; Figures 4F, 4G, S5D, and S5E). These cells resembled DN B cells lacking *CD27* and *CR2* but retained low *IGHD* and *CXCR5* transcription—likely a mix of GCB-like and DN-like CD69^+^ cells seen by CyTOF (clusters 14 and 1; Figure S3A).

CD69 expression was higher across all infection-induced B cells in adults (Figure 4I). In contrast, infected children showed increased transitional B cells (cluster 5, *CD24*^*+*^*MME*^*+*^*CD38*^*+*^*CR2*^*+*^) and IFN-responsive naive B cells (cluster 4, *IGHD*^*+*^*IGHM*^*+*^*CR2*^*+*^*ISG*^*+*^), matching CyTOF-identified transitional and naive-like populations (clusters 3 and 5; Figure S3B).

Cluster 4 (ISG^+^, GCB-like naive) also showed high CXCR5 and likely included GCB-like B cells seen in CyTOF (cluster 11). An additional feature in children was *IL3RA* (CD123) expression, absent in adult B cells. Plasmablast frequencies were similar across age groups.

A more detailed comparison of B cells from infected children and adults accumulating in the clusters described earlier revealed further important transcriptional differences (Figure 4H). First, the ISG signature of B cell cluster 4 was much more pronounced in samples of infected children, whereas expression of lead genes (*CD83*, *CD69*, and *TNFRSF13C*, encoding for the BAFF receptor) within DN-like, activated, *CD69*^high^ B cells (cluster 6) was increased in adults. Expression of *CD83* and *CD69* was higher in adults compared to children when analyzing the total number of B cells contained in all infection-induced clusters (Figure 4I) highlighting the concordance with the CyTOF (Figure S3F) data. Plasmablasts from infected adults showed little *ISG15* expression but prominent *SOCS3* transcription (Figure 4H). Those transcriptional differences between plasmablasts of infected children and adults could indicate diverging differentiation routes.

Indeed, analyzing the clonal overlap of B cell receptors between infection-induced B cell populations and plasmablasts revealed that children exclusively generated plasmablasts from transitional and ISG^+^ B cells, whereas both DN and DN-like, activated, CD69^high^ B cell populations in addition to ISG^+^ B cells contributed to the plasmablast pool in infected adults (Figure 4J). This might explain the enhanced *SOCS3* transcription, which is known to be increased in CD21^−^ DN B cells due to *STAT3* overactivation.^35^

### Consequences for local T cell activation and antibody profiles

To establish a link between the observed systemic age-dependent differences and local responses, we reanalyzed T cells from our previously published nasal swab scRNA-seq data from samples of infected patients and controls.^8^ Subclustering of TCRab^+^ T cells resulted in 8 main clusters (Figure S6A). Like PBMC TCRab^+^ T cells, the cells were classified based on the coreceptor transcription as CD4^+^ or CD8^+^ and broadly categorized using established T cell differentiation markers into naive, CM, EM, and CTLs (Figure S6A). The information of the top 10 differentially expressed markers in each cluster was used to further annotate the populations according to their cluster-specific mRNA expression patterns (full cluster-specific marker list in Table S4).

**Figure 5:**
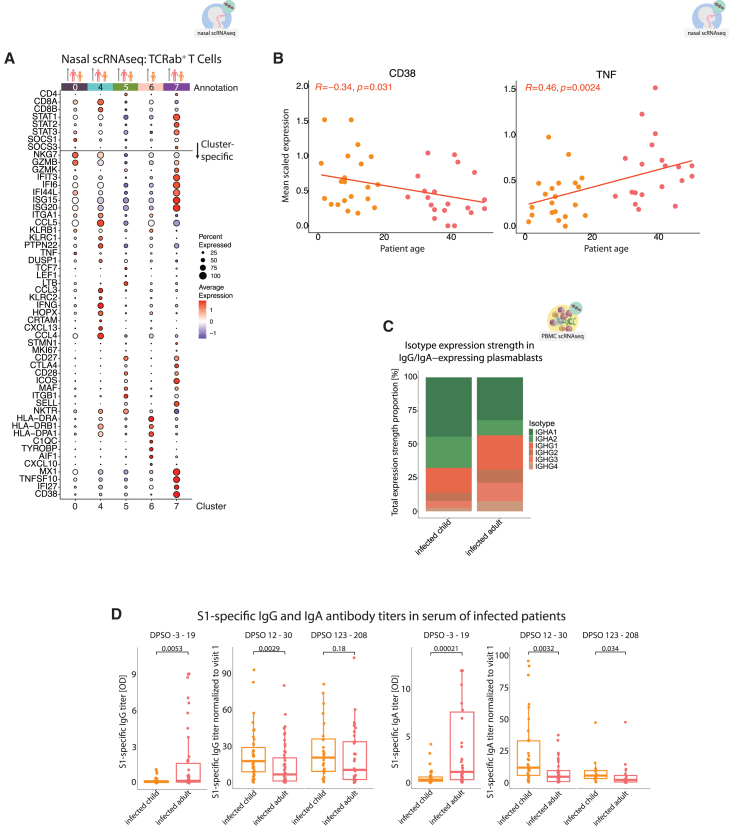
Consequences for local T cell responses and generated antibody profiles (A) Dotplot, showing scaled average expression of genes in TCRab^+^ T cells, subset from the nasal swab scRNA-seq data.^8^ Clusters, increased with infection (0, 4, 5, 6, and 7). A total of 8 clusters have been produced using a graph-based approach as implemented in Seurat package (KNN graph with Louvain community detection). A horizontal line splits the dotplot in two parts; genes above the line were curated based on the presence of clusters with pronounced ISG signature and include other genes useful for annotation; genes below the line were found to be differentially expressed between the clusters (FindMarkers Seurat function). (B) Scatterplots showing CD38 and TNF genes transcription (average scaled expression in clusters 0, 4, 5, 6, and 7, expanded with infection) for each donor, plotted against donor’s age, using TCRab^+^ T cells, subset from nasal swab scRNA-seq data.^8^ Linear models fitted to the data points and Spearman’s rank correlation coefficients. (C) Stacked bar chart showing relative expression strength of heavy-chain genes encoding for the different IgG and IgA isotypes in plasmablasts (B cell cluster 12, PBMC scRNA-seq experiment). Plasmablasts, expressing either of the heavy-chain genes, were pre-selected. Expression values for each gene were calculated and normalized to the total expression of all heavy-chain genes. (D) Boxplots of S1-specific IgG (left) and IgA (right) antibody titers for the acute infection phase and follow-up measurements done approximately 2 weeks and 6 months later. Titers for second and third time points are normalized to the first time point for each patient (fold change and ratio). Wilcoxon *p* values.

From those, T cell cluster 6 was only enriched in infected children, whereas clusters 0, 4, 5, and 7 were enriched in both infected children and infected adults (Figures 5A and S6B). Clusters 0 and 7 were ISG^+^ CD8^+^ and CD4^+^ T cell clusters, respectively (Figures 5A and S6A). Transcription of IFN response genes and especially of *CD38* was higher in cluster 7 T cells from infected children in comparison to infected adults (Figure 5A). In contrast, cluster 0 and 4 T cells from infected adults showed increased *TNF* gene expression. Consequently, plotting mean scaled expression of *CD38* and *TNF* in infection-induced expanded clusters against age revealed significant correlations. Similar to the systemic response shown before (Figure 2D), we detected a negative correlation of *CD38* expression in mucosal T cells with age, whereas proinflammatory *TNF* transcription gradually increased with age (Figure 5B).

Next, we studied whether the diverse plasmablast differentiation programs were linked with age-dependent differences in antibody responses. Transcriptional analysis of Ig heavy-chain genes in plasmablasts revealed an increased relative expression of IgA isotypes in samples of children in comparison to those from adults, which had higher expression of IgG and especially complement-fixing IgG1 and IgG3 isotypes (Figure 5C).

Studying antibody responses in serum of infected patients, SARS-CoV-2-infected adults showed a swift anti-S1 IgA and IgG antibody response during the first 2 weeks after symptom onset (visit 1, DPSO −3–19; Figure 5D), which was significantly enhanced, compared to infected children. Although children responded with a delay, we detected a strong increase of IgA production at later time points (visits approximately 2 weeks after the first, DPSO 12–30, and approximately 6 months after the first, and DPSO 123–208; Figure 5D).

### Age-dependent immune cell activation profiles are independent of comorbidities and conserved in a hamster infection model

Different risk factors have been established for COVID-19 severity.^36^ In order to exclude comorbidities as driving factors of the observed age-dependent phenotypes, we tested for confounding effects using linear regression modeling.

Using data from the CyTOF measurements, we displayed results from the CD4^+^ and B cell profiling of SARS-CoV-2-infected patients in a scatterplot (Figure S6C). Importantly, the presence of comorbidities and/or obesity did not significantly alter the linear correlation between age and CD38 and CCR6 expression by CD4^+^ T cells or CXCR5 and CD69 expression by B cells, indicating that the differences that we identified in our study are indeed driven by age and not a function of confounding comorbidities.

Importantly, patient stratification according to disease severity showed that, even when comparing symptomatic children to mildly affected adults, the age-dependent patterns of infection-induced monocyte, CD4^+^, and B cell subsets remained (see Mendeley Figure 2). However, the samples from the severely affected patients showed a more pronounced phenotype.

To further substantiate our findings, we used samples from a SARS-CoV-2 hamster model, which also shows age-dependent infection severity.^37^ We analyzed bulk RNA sequencing of whole blood from young (6 weeks) and old (32–34 weeks) infected hamsters and uninfected controls.^37^ We tested whether the human gene sets could distinguish young from old infected hamsters, and, indeed, they clustered separately (Figure S6D). Young hamsters showed elevated ISGs (*Isg15*, *Oas3*, *Ifit1*, and *Irf7*), while old ones had higher *Il1b* and *S100* gene expression, closely resembling transcription profiles of human monocytes and T cells (Figures 3C, 3D, 4C, and 4D). None of these transcripts were present in uninfected samples. These results confirm age-dependent immune responses to SARS-CoV-2 in a controlled experimental system without comorbidity-related confounding.

### Disturbed type I IFN responsiveness reduces formation of CD38^+^ CD4^+^ T cells and enhances cytokine production in older individuals

**Figure 6:**
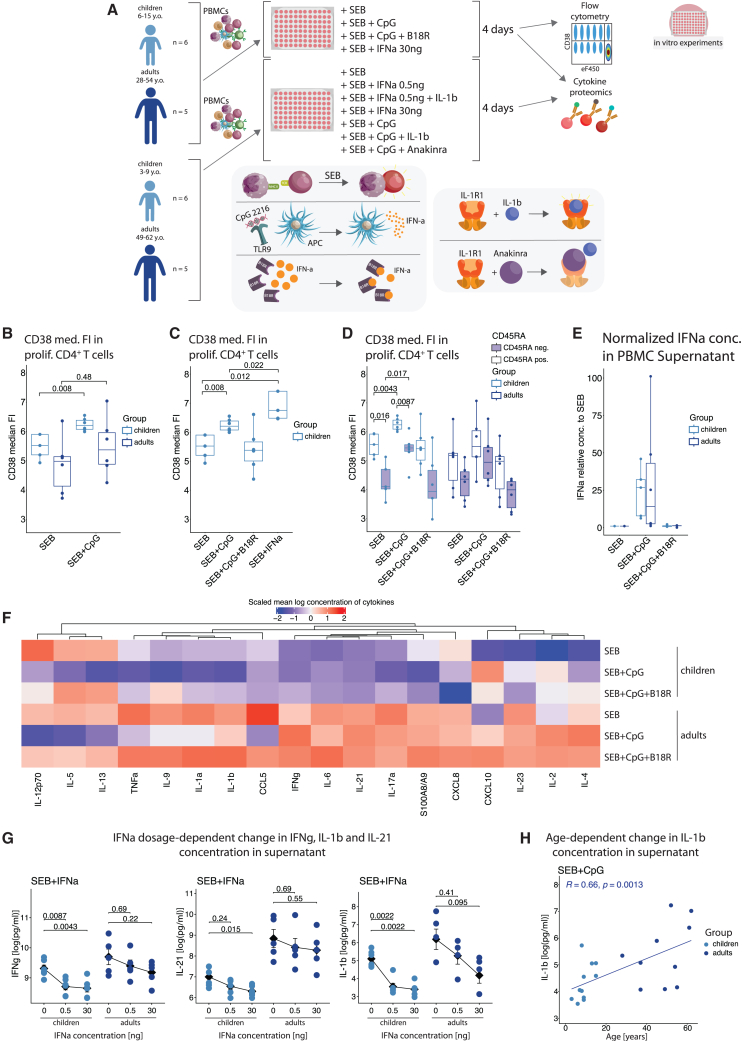
Mechanistic *in vitro* studies link age-dependent rewiring of type I IFN responsiveness with *in vivo*-detected opposite activation profiles (A) Overview of the workflow used to study the responsiveness to type I IFN and IL-1b. PBMCs from uninfected children and adults were stimulated with either SEB or a combination of SEB, ODN CpG2216, B18R, and recombinant IFNa. In a parallel experiment series, different concentrations of IFNa as well as combinations of SEB, ODN CpG2216, IL-1b, and IL-1b inhibitor anakinra were tested. After 4 days of incubation, phenotypic differences in activation marker expression were determined by flow cytometry, while cell culture supernatants were used for cytokine and chemokine quantification. Experiments focused on IL-1b and anakinra influence were only measured in cytokine proteomics. (B) Boxplot of arcsinh-transformed median CD38 fluorescence intensity in proliferating CD4^+^ T cells, showing influence of CpG2216-mediated activation on CD38 expression. Wilcoxon test *p* values. Dropout in uninfected children group SEB condition is due to low cell number. (C) Boxplot of arcsinh-transformed median CD38 fluorescence intensity in proliferating CD4^+^ T cells, showing influence of CpG2216-mediated activation and IFNa (30 ng/mL) on CD38 expression in children. Wilcoxon test *p* values. Dropouts in SEB and SEB+IFNa perturbations are due to low cell number. (D) Boxplots of CD38 median signal intensity in proliferating CD4^+^ T cells, separated into CD45RA^−^ (violet filling) memory and CD45RA^+^ naive subpopulations, showing the difference in CD38 upregulation in response to CpG2216-mediated activation and IFNa release between memory and naive CD4^+^ T cells. Wilcoxon test *p* values. Dropout in uninfected children, SEB perturbation is due to low cell number. (E) Boxplot of IFNa concentration measured in cell culture supernatant and normalized to values detected in SEB condition for each patient, showing the effectiveness of CpG2216 in provoking IFNa release as well as of B18R in reducing the concentration of soluble IFNa. Dropout in uninfected children is due to low cell number. (F) Heatmap, showing scaled average log concentration of the 18 cytokines measured in co-culturing experiments for different perturbations using PBMC. (G) Line plots, showing the dependence of IFNg, IL-21, and IL-1b concentrations on the IFNa concentration. Wilcoxon *p* values. (H) Scatterplot, illustrating the correlation between donor age and IL-1b concentration in supernatant when PBMCs are stimulated with SEB and CpG.

To test whether divergent T cell phenotypes in children and adults reflect different type I IFN responsiveness, we cultured PBMCs from uninfected donors of both age groups (Figure 6A). Cells were stimulated with Staphylococcal enterotoxin B (SEB) as a TCR stimulus, with or without CpG2216—an oligonucleotide that activates TLR9 and induces IFNa, mimicking viral infection.^38^^,^^39^

CpG increased CD38 expression on CD4^+^ T cells, but significantly only in children (Figure 6B). This upregulation was reproduced by recombinant IFNa and blocked by the type I IFN decoy receptor B18R (Figure 6C). Notably, the effect was independent of naive/memory T cell proportions; both compartments in children, but not in adults, significantly upregulated CD38 (Figure 6D). CpG triggered similar IFNa levels in PBMCs from both groups, suggesting altered responsiveness rather than production differences (Figure 6E). Thus, activated T cells in adults exhibit age-related resistance to type I IFN.

CD38 depletes nicotinamide adenine dinucleotide (NAD), limiting T cell activation.^40^ Its enzymatic role boosts regulatory T cell (Treg) activity while suppressing cytotoxic, Th1, and Th17 functions.^41^^,^^42^^,^^43^ Type I IFN also modulates T cell cytokine output, notably IL-17A and IL-21.^44^^,^^45^^,^^46^ Therefore, age-related IFN responsiveness likely affects inflammatory cytokine regulation.

We tested this by stimulating PBMCs with SEB and CpG and profiling 18 cytokines, chemokines, and S100A8/A9, followed by unsupervised clustering (Figure 6F), leading to detection of four clusters:

In the first cluster, IL-2, IL-4, IL-23, and CXCL10 increased with CpG in both age groups.

In the second cluster, IL-12p70, IL-5, and IL-13 were equally induced by SEB but suppressed by CpG. The suppression was IFN dependent and could be reversed by B18R.

In the third cluster, IL-17A, IL-6, S100A8/A9, IFNg, IL-21, and CXCL8 were also reduced by CpG, but levels were slightly higher in adult cultures (IL-17A *p* = 0.015, IL-6 *p* = 0.049, S100A8/A9 *p* = 0.041, IFNg *p* = 0.066, IL-21 *p* = 0.11, and CXCL8 *p* = 0.27). Notably, CpG increased IFNg and IL-21 only in adults but reduced S100A8/A9 and CXCL8 only in children—mirroring CD38 patterns (Figures 6B–6D). Dose-response analysis confirmed that IFNg and IL-21 were more IFNa sensitive in children (Figure 6G).

In the fourth cluster, IL-1b, IL-1a, IL-9, and TNF were more abundant in adult cultures (IL-1b *p* = 0.006, IL-1a *p* = 0.012, IL-9 *p* = 0.049, and TNF *p* = 0.066). Even small amounts of IFNa significantly reduced IL-1b in children’s samples but not adults’ (Figure 6G). IL-1b, TNF, and IL-9 levels also positively correlated with age (Figure 6H; TNF R = 0.47, *p* = 0.032; IL-9 R = 0.45, *p* = 0.038).

Type I IFN and IL-1 are known antagonists.^47^^,^^48^ To test whether IL-1 counteracts IFN-mediated cytokine suppression, we added IL-1 to cultures with type I IFN (Figure 6A, lower cohort). IFNa alone suppressed IL-6, IL-9, and IL-17A (Figure S7), but co-addition of IL-1b restored their production. Conversely, blocking IL-1 with anakinra reduced IL-17A in SEB+CpG cultures, while IL-1b addition enhanced IL-6, IL-9, and IL-17A. No such IL-1-IFN interplay was seen for IL-5.

Higher *in vitro* cytokine levels in adult PBMCs mirrored *in vivo* data showing elevated IL-1b, IL-6, CXCL8, and TNF in serum from infected adults (Figure S4G), upregulated S100A8/A9 in adult monocytes and CD4^+^ T cells (Figures 3D and 4D), and increased TNF in nasal T cells (Figure 5B).

### Enhanced STAT3 activation characterizes IFNa signaling in T and B cells during aging

**Figure 7:**
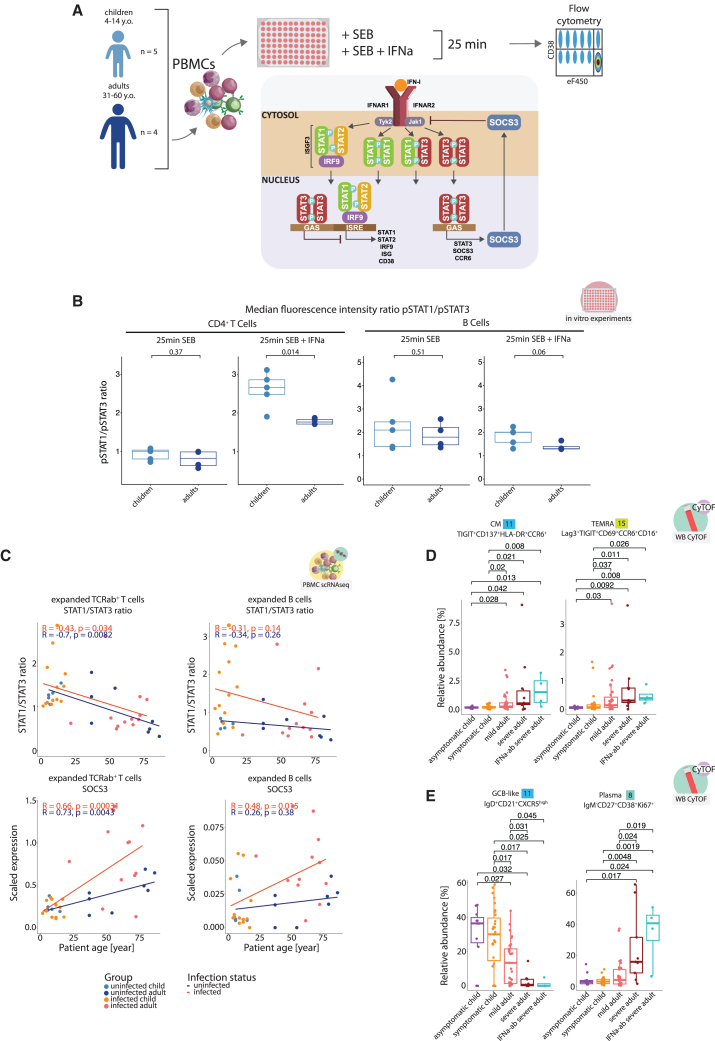
Altered type I IFN signaling and gradual involvement of STAT3 activation in stimulated T and B cells of older individuals (A) Overview of the workflow studying the phosphorylation dynamics of STAT1 and STAT3 in T cells and B cells from children and adults. PBMCs from control children and adults were stimulated with either SEB or a combination of SEB and recombinant IFNa. After 25 min of incubation, levels of phosphorylated STAT1 and STAT3 were determined by flow cytometry for pre-gated populations. (B) Boxplots, showing the ratio of pSTAT1 to pSTAT3 (based on measured median fluorescence intensity) for CD4^+^ T cells and B cells of control children and adults following 25-min incubation with SEB or SEB and IFNa. Wilcoxon *p* value. (C) Scatterplots of STAT1/STAT3 transcription ratio and SOCS3 transcription (average scaled expression in clusters expanded with infection) for each donor, plotted against donor’s age, using PBMC scRNA-seq experiment data (details in Table S1). CD4^+^ T cells were a subset of the total TCRab^+^ pool using CD8A and CD8B genes (assigned CD4 FLAG, if cell is negative for both). Spearman’s rank coefficients. (D) Boxplots showing relative abundance of infection-induced clusters 11 and 15 from the FlowSOM algorithm, calculated per sample within all CD4^+^ T cells from the CyTOF data. Wilcoxon *p* values. (E) Boxplots, showing relative abundance of infection-induced clusters 8 and 11 resulting from the FlowSOM algorithm, calculated per sample within all B cells from the CyTOF data. Wilcoxon *p* values.

Our transcriptional analyses indicated a shift from a predominant STAT1 to STAT3 activity in T and B cells from SARS-CoV-2-infected adults. To confirm the altered balance, we determined the amount of phosphorylated STAT1 (pSTAT1) and STAT3 (pSTAT3) by flow cytometry in the *in vitro* culture system (Figures 7A and 7B). SEB stimulation alone did not lead to an altered balance of STAT1 and STAT3 activation in CD4^+^ T and B cells from children and adults (Figure 7B). Addition of IFNa led to an increase in the pSTAT1 to pSTAT3 ratio in PBMC cultures from children. In contrast, adult cells responded to IFNa stimulation with enhanced STAT3 activation maintaining a lower pSTAT1/pSTAT3 ratio.

Interestingly, the differences in the balance between STAT1 and STAT3 involvement were also apparent *ex vivo* as we observed an age-dependent gradual decline in the ratio of *STAT1*/*STAT3* transcription for infection-induced (expanded) CD4^+^ T cells as well as B cell populations (Figure 7C). Consequently, *SOCS3*, as a STAT3 target gene, followed an opposite pattern.

To functionally validate the importance of type I IFN signaling for the disparate phenotypes of CD4^+^ T and B cells during SARS-CoV-2 infection, we investigated samples from individuals with neutralizing antibodies to type I IFN (Figures 7D and 7E). Indeed, both CD4^+^ T cells and B cells from patients with autoantibodies to IFN showed an extreme “aged” phenotype, characterized by the highest median abundance of both adult-specific CD4^+^ T cell populations (CyTOF cluster 11 TIGIT^+^CD137^+^HLA-DR^+^CCR6^+^ CM and cluster 15 Lag3^+^TIGIT^+^CD69^+^CCR6^+^CD16^+^ TEMRA), lowest median abundance of GCB-like cells (CyTOF cluster 11 IgD^+^CD21^+^CXCR5^high^), and highest proportion of plasmablasts in total B cells (CyTOF cluster 8 IgM^−^CD27^+^CD38^+^Ki67^+^) among the infected patient samples.

In summary, the altered T and B cell phenotypes in samples from infected adults could be linked to a gradual loss of canonical type I IFN signaling via STAT1 and a shift toward STAT3 signaling.

## Discussion

Different clinical manifestations of viral respiratory infections across age groups are well known but poorly understood.^10^ Before SARS-CoV-2, comparing immune responses across ages was challenging due to pre-existing immunity in adults. Using a unique patient cohort, we systematically compared innate and adaptive immune responses to the same primary infection in children and adults.^5^ Our study reveals distinct immune profiles and a shift from STAT1/STAT2-mediated antiviral ISG responses to STAT3-driven inflammatory response across immune cell types.

Alongside *in vitro* evidence, we show an age-associated rewiring of type I IFN signaling, shifting from canonical IFN responses to proinflammatory STAT3 signaling, most prominent after age 55, mirroring the age-dependent risk of severe COVID-19. In children, strong ISG induction was linked to HLA-DR^high^ monocytes and follicular T/B cell responses that favored IgA-expressing plasmablasts. In contrast, aging led to HLA-DR^low^, CXCL8- and S100A8/A9-expressing monocytes, CCR6^+^CD69^+^ T helper cells, and atypical B cells, resulting in faster, complement-binding IgG-dominated responses.

We confirmed prior findings of reduced circulating pDCs with age but showed that IFNa production remains intact in older adults, suggesting altered responsiveness, rather than diminished production, underlies age-related immune differences. We explored whether IFN signaling itself was rewired. Type I IFN binding activates JAK1 and TYK2, triggering STAT1/2 phosphorylation and ISGF3-mediated ISG transcription.^49^ However, type I IFNs can also activate STAT3, which may dampen canonical signaling by sequestering STAT1 or inducing negative regulators such as *SOCS1* and *SOCS3*, thereby modulating the cell’s responsiveness to type I IFNs.^29^^,^^30^^,^^31^^,^^32^^,^^49^^,^^50^^,^^51^ Thus, the balance between STAT1/STAT2 versus STAT3 engagement determines the signaling output of type I IFN signaling. Our data show diminished STAT1 activation and increased STAT3 engagement in older adults.

Aging-associated effects like failure to exclude SHP-1 from the JAK/STAT complex to ensure sustained STAT1 signaling and enhanced STAT3 activation in CD8^+^ T cells leading to severe simian immunodeficiency virus infections have been previously described.^52^^,^^53^ The central role of the aberrant STAT signaling in COVID-19 pathology is also a known concept.^54^ We provide evidence that it is not limited to infected cells but that the aging-related skew toward STAT3 also applies to cell of the immune system.

Extracellular modulators also matter. IFN and IL-1 counter-regulate one another.^47^ We found higher IL-1b levels in adult serum and PBMC cultures post-infection, correlating with age and promoting IL-6, IL-9, and IL-17A secretion when combined with IFNa. We also demonstrated a mutually negative activity of IFNa and IL-1b, where IL-1b enhanced secretion of proinflammatory cytokines IL-6, IL-9, and IL-17A.

STAT3 overactivation was linked to transcriptional changes in monocytes, CD4^+^ T cells, and B cells during aging. In line with our previous findings, SARS-CoV-2-induced monocytes in adults showed low HLA-DR expression, consistent with STAT3-mediated major histocompatibility complex class II downregulation, unlike the IFN-driven, activated HLA-DR^high^ profiles seen in children.^17^^,^^30^^,^^55^^,^^56^ Adult monocytes also showed enriched transcription of CXCL8, CXCL2, and S100A8/A9/12—known STAT3 targets.^57^^,^^58^^,^^59^^,^^60^

Supporting this, aged hamsters with SARS-CoV-2 showed decreased ISG expression and increased *Il1b* and *S100* induction, suggesting qualitative immune changes rather than simple expression shifts.^61^ STAT1/2 vs. STAT3 engagement in CD4^+^ T cells correlated with CD38 and CCR6 expression, respectively. CD38, an NAD-consuming enzyme, enhances Treg activity and dampens Th1/Th17 responses, potentially explaining low inflammatory gene expression in children’s T cells and faster immune contraction.^40^^,^^41^^,^^42^^,^^43^

STAT3 overactivation favors differentiation of CD21^low^CCR6^+^ atypical B cells—key features of adult SARS-CoV-2 responses.^35^ These extrafollicular responses, previously linked to disease severity, are promoted by increased peripheral T helper cells in adults.^23^^,^^26^^,^^62^^,^^63^

Here, we link this phenomenon to age-induced IFN signaling deviation.

Children’s plasmablasts preferentially produced IgA isotypes, while adults’ primarily generated complement-fixing IgG isotypes, enhancing immune complex formation and classical complement activation.^64^^,^^65^ This is in line with our previous findings, where we showed a direct link between complement activation and T cell-driven immunopathology in adult COVID-19 patients.^18^

Overall, we demonstrate that age-induced IFN signaling rewiring alters orchestration of immune cell activation. Children’s STAT1/STAT2-dominant responses are protective, while aging shifts the balance toward STAT3, promoting hyperinflammatory, pathogenic states. This signaling switch, also demonstrated *in vitro* and in animal model, appears to be a general phenomenon. Future work should explore its impact on immunization responses and guide age-tailored regimens.

### Limitations of the study

We conducted an observational cohort study comparing immune responses of children and adults to acute SARS-CoV-2 infection. While observational studies are limited by potential confounders and lack causal inference, we took extensive measures to minimize bias and enhance comparability. To reduce environmental factors, some children and adults were recruited from the same households. We also accounted for comorbidities and obesity by modeling their effects on key findings.

Beyond study design constraints, generalizability may be limited, as most adult infections with other viruses occur in immune-experienced individuals, unlike the naive hosts studied here.

## Resource availability

### Lead contact

Further information and requests for resources and reagents should be directed to the lead contact, Birgit Sawitzki (birgit.sawitzki@bih-charite.de).

### Materials availability

This study did not generate new unique reagents.

### Data and code availability

• This study has generated data in standardized formats and custom code for processing and analysis of CyTOF and scRNA-seq data, which can be found in repositories listed in the following.
• Debarcoded, batch-corrected, and pre-gated fcs files as well as debarcoded, non-normalized, and non-pre-gated fcs files of the CyTOF experiment are deposited at Figshare: https://doi.org/10.6084/m9.figshare.26131711.v1. Pre-processed (demultiplexed, integrated, quality-controlled, and merged with metadata) scRNA-seq data are deposited in rds format at Figshare: https://doi.org/10.6084/m9.figshare.26131711.v1. Raw count data of the scRNA-seq experiment are deposited at GEO repository under the accession ID GEO: GSE271284 (https://www.ncbi.nlm.nih.gov/geo/query/acc.cgi?acc=GSE271284).
• R-scripts to analyze the CyTOF and single-cell data are available in Mendeley Data: https://doi.org/10.17632/kz7cpw3bnt.1.
• Any additional information required to reanalyze the data reported in this work paper is available from the lead contact upon request.

## Acknowledgments

We thank Desireé Kunkel and Jacqueline Keye from the BIH Flow and Mass cytometry Core facility for the help with cytometry data generation, the BIH/MDC Genomics Platform for sequencing, Benedikt Obermayer-Wasserscheid for pre-processing of the sequencing data, and the Clinical Study Center (CSC) at the Berlin Institute of Health (BIH) and the Central Biobank of the BIH (ZeBanC) for ongoing support of the PA-COVID-19 and RECAST studies.

We are grateful to the patients and donors volunteering to participate in this study, making this research possible in the first place.

This work was supported by the German Research Foundation (DFG) SA1383/3-1 and CRC 1444 - project 427826188 to B.S.; 322900939, 454024652, and 432698239 to P.B.; CRC 1449 - project 431232613 to M.A.M.; SFB-TR84 114933180 to L.E.S.; the German Federal Ministry of Education and Research (BMBF) NATON, no. 01KX2121, to P.B., B.O., and B.M.; 82DZL009C1 and 01GL2401A to M.A.M.; 01IK20337 - RECAST to B.S., J.R., M.A.M., V.M.C., L.E.S., and M.R.; FKZ 01KX2021 - COVIM to B.S., L.E.S., and V.M.C.; 01GM2202C to P.B.; VARIPath (01KI2021) to V.M.C.; the European Research Council: 825392 - RESHAPE to B.S.; ERC Consolidator Grant AIM.imaging.CKD, no. 101001791, to P.B.; the Jürgen Manchot Foundation to A.H.; and a Charite' 3R project to B.S.

## Author contributions

Conceptualization: M.R., M.A.M., V.M.C., L.E.S., J.R., and B.S.; methodology: L.P., S. Brumhard, P.G., R.A.-G., N.B., C.I., R.D.B., T.M., and V.M.C.; software/formal analysis: L.P., P.G., S.v.S., V.M.C., and B.S.; investigation: L.P., S. Brumhard, P.G., A.H., K.V., E.W., H.-P.D., S.v.S., L.M., and T.M.; resources: S.W., P.G., A.H., B.M., C.L., J.L., R.E., I.L., B.O., J.T., P.B., M.A.M., V.M.C., and J.R.; data curation: L.P., S.W., S.v.S., and J.L.; writing – original draft: L.P., S.v.S., S. Bedoui, C.M., and B.S.; writing – review and editing: L.P., P.G., S.v.S., R.D.B., I.L., P.B., S. Bedoui, V.M.C., L.E.S., J.R., and B.S.; visualization: L.P., S.v.S., and B.S.; supervision: C.I., R.D.B., P.B., L.E.S., J.R., and B.S.

## Declaration of interests

V.M.C. is named together with Charite' and Euroimmun GmbH on a patent application on the diagnostic of SARS-CoV-2 by antibody testing.

## STAR★Methods

### Key resources table

**Table:** 

REAGENT or RESOURCE	SOURCE	IDENTIFIER
**Antibodies**		

89Y_CD45_Hi30	Standard BioTools	Cat#3089003B, RRID:AB_2938863
103Rh_live/dead	in-house	N/A
104Pd_β2M_2M2	Biolegend	Cat# 316302; RRID: AB_492835
106Pd_β2M_2M2	Biolegend	Cat# 316302; RRID: AB_492835
108Pd_β2M_2M2	Biolegend	Cat# 316302; RRID: AB_492835
110Pd_β2M_2M2	Biolegend	Cat# 316302; RRID: AB_492835
113In_HLADR_L243	Biolegend	BioLegend Cat# 307651, RRID:AB_2562826
115In_CD3E_UCHT1	Biolegend	Cat# 300443, RRID:AB_2562808
141Pr_CD196 (CCR6)_G034E3	Standard BioTools	Cat#3141003A, RRID:AB_2687639
142ND_CD19_HIB-19	Standard BioTools	Cat#3142001B, RRID:AB_3661857
143ND_CD123 (IL-3RA)_6H6	Standard BioTools	Cat#3143014B, RRID:AB_2811081
144ND_CD15 (FUT4)_W6D3	Standard BioTools	Cat#3144019B, RRID:AB_2892685
145ND_CD21_HB5	Biolegend	Cat#1380
146ND_CD226_REA1040	Miltenyi	Cat# 130-126-485, RRID:AB_2889512
147Sm_IgD_IgD26	Biolegend	Cat#330401
148ND_ICOS_C398.4A	Standard BioTools	Cat#3148019B, RRID:AB_2756435
149Sm_CD96_REA195	Miltenyi	Cat# 130-101-039, RRID:AB_2659661
150ND_KLRG1_REA261	Miltenyi	Cat# 130-103-751, RRID:AB_2652582
151Eu_TCRgd_11F2	Miltenyi	Cat# 130-096-862, RRID:AB_2654074
152Sm_CD95 (FAS)_DX2	Biolegend	Cat#355
153Eu_TIGIT_MBSA43	Standard BioTools	Cat#3153019B, RRID:AB_2756419
153Eu_CD62L (L-Selectin)_DREG56	Biolegend	Cat# 304835, RRID:AB_2563758
155Gd_CD27_L128	Standard BioTools	Cat#3155001B, RRID:AB_2687645
156Gd_CXCR3_G025H7	Standard BioTools	Cat#3156004B, RRID:AB_2687646
157Gd_KLRF1_REA845	Miltenyi	Cat# 130-126-475, RRID:AB_2889721
158Gd_CD10_HI10a	Standard BioTools	Cat#3158011B, RRID:AB_2921314
160Gd_CD14_RMO52	Standard BioTools	Cat# 3160006, RRID:AB_2661801
161Dy_CD28_L293	BD	Cat# 348040; RRID:AB_400367
162Dy_CD69_FN50	Standard BioTools	Cat#3162001B, RRID:AB_3096016
163Dy_CD294 (CRTH2)_BM16	Standard BioTools	Cat#3163003B, RRID:AB_2810253
164Dy_CXCR5_51505	Standard BioTools	Cat# 3164016B; RRID: AB_2687858
166Er_CD34_581	Standard BioTools	Cat#3166012B, RRID:AB_2756424
167Er_CD38_HIT2	Standard BioTools	Cat#3167001B, RRID:AB_2802110
168Er_MKI67_Ki-67	Standard BioTools	Cat# 3168007B; RRID: AB_2800467
169Tm_CD25_2A3	Standard BioTools	Cat# 3169003; RRID: AB_2661806
170Er_LAG3 (CD223) _11C3C65	Biolegend	Cat# 369302; RRID: AB_2616876
171Yb_KLRB1 (CD161)_HP-3G10	Biolegend	Cat# 339919; RRID: AB_2562836
172Yb_CD45RO_4G11	in house/DRFZ	ID#5788
173Yb_CD137_4B4-1	Standard BioTools	Cat#3173015B, RRID:AB_3675476
175Lu_PD-1_EH12.2H7	Standard BioTools	Cat# 3175008; RRID: AB_2687629
176Yb_CD56 (NCAM)_NCAM16.2	Standard BioTools	Cat# 3176008; RRID: AB_2661813
191Ir_DNA	Standard BioTools	N/A
193Ir_DNA	Standard BioTools	N/A
194Pt_CD8A_GN11	in house/DRFZ	Cat#925
195Pt_IgM_MHM-88	Biolegend	Cat# 314502; RRID: AB_493003
196Pt_CD11c (ITGAX)_BU15	Biolegend	Cat# 337221, RRID:AB_2562834
198Pt_β2M_2M2	Biolegend	Cat# 316302; RRID: AB_492835
209Bi_CD16 (FCGR3A)_3G8	Standard BioTools	Cat#3209002B, RRID:AB_2756431
CD3 - BUV496	BD	Cat# 612940, RRID:AB_2870222
PD1 - BUV805	invitrogen	Cat# 368-9985-82
CD8 - BV510	Biolegend	Cat# 301048, RRID:AB_2561942
CD16 - BV605	Biolegend	Cat# 302040, RRID:AB_2562990
CD27 - BV650	Biolegend	Cat# 302828, RRID:AB_2562096
CXCR5 - BV711	Biolegend	Cat# 356934, RRID:AB_2629526
HLADR - BV785	Biolegend	Cat# 307642, RRID:AB_2563461
CRTH2 - PE	Biolegend	Cat# 350106, RRID:AB_10900060
GranzymeB - ECD	in house	in house
CD38 - PE cy7	Biolegend	Cat# 356608, RRID:AB_2561904
ld - ZombieRed	Biolegend	Cat# 67685
Lag3 - PerCpef710	invitrogen	Cat# 46-2231-82
CCR6 - APC	Biolegend	Cat# 353416, RRID:AB_10915987
CD4 APC-Fire 750	Biolegend	Cat# 300560, RRID:AB_2629693
BD Phosflow™ Alexa Fluor® 488 Mouse Anti-Stat1 (pY701), Clone 4a	BD Biosciences	Cat# 612596, RRID: AB_399879
BD Phosflow™ PE Mouse Anti-Stat3 (pY705), Clone 4/P-STAT3	BD Biosciences	Cat# 562072, RRID: AB_10893601
APC/Cyanine7 anti-human CD4 Antibody, Clone RPA-T4	BioLegend	Cat# 300518, RRID:AB_314086
eBioscience™ Fixable Viability Dye eFluor™ 506	Thermo Fisher Scientific	Cat# 65-0866-14
CD19 Pacific Blue	BioLegend	Cat# 302232, RRID:AB_2073118
BD Horizon™ BUV737 Mouse Anti-Human CD8, Clone SK1	BD Biosciences	Cat# 612754, RRID:AB_2870085
BD Horizon™ BUV496 Mouse Anti-Human CD3, Clone UCHT1	BD Biosciences	Cat# 612940, RRID:AB_2870222

**Biological samples**		

Human blood	Charité Berlin	N/A

**Chemicals, peptides, and recombinant proteins**		

Staphylococcal enterotoxin B (SEB)	Sigma	S4881-1MG
hIFN-α 2a	Shenandoah	100–54
B18R	R&D Systems, Biotechne	8185-BR-025
CD38 Inh_78c	Tocris, Biotechne	6391

**Critical commercial assays**		

Simoa CORPLEX™ CYTOKINE PANEL (IFN-γ, IL-1β, IL-4, IL-5, IL-6, IL-8, IL-10, IL-12P70, IL-22, TNFα)	Quanterix	Cat# 85-0329
Cytokine/Chemokine/Growth Factor 45-Plex Human ProcartaPlex™ Panel 1	Invitrogen, Thermo Fisher Scientific	Cat# EPX450-12171-901
18-Plex Human ProcartaPlex™ Mix&Match panel (custom, IFN-γ, IL-1a, IL-1b, IL-12p70, IL-13, IL-17aA, IL-2, IL-21, IL-23, IL-4, IL-5, IL-6, IL-8, IL-9, IP-10, RANTES, S100A8/A9, TNFa)	Invitrogen, Thermo Fisher Scientific	Cat# EPX450-12171-901
Chromium Next GEM Chip G Single Cell Kit	10x Genomics	Cat# 1000120
Chromium Next GEM Single Cell 5′ Library and Gel Bead Kit v1.1	10x Genomics	Cat# 1000167
Chromium Single Cell 5′ Library Construction Kit	10x Genomics	Cat# 1000020
Chromium Single Cell V(D)J Enrichment Kit, Human B Cell	10x Genomics	Cat# 1000016
Chromium Single Cell V(D)J Enrichment Kit, Human T cell	10x Genomics	Cat# 1000005
Single Index Kit T Set A	10x Genomics	Cat# 1000213
Single Index Kit N Set A	10x Genomics	Cat# 1000212
Chromium Single Cell 5′ Feature Barcode Library Kit	10x Genomics	Cat# 1000080
Qubit 1X dsDNA HS Assay Kit	Thermo Fisher Scientific	Cat# Q33231
High Sensitivity DNA Kit	Agilent	Cat# 5067-4626

**Deposited data**		

WB CyTOF Monocytes & DCs	This paper	Figshare: https://doi.org/10.6084/m9.figshare.26131711.v1
WB CyTOF CD4^+^ T Cells	This paper	Figshare: https://doi.org/10.6084/m9.figshare.26131711.v1
WB CyTOF B Cells	This paper	Figshare: https://doi.org/10.6084/m9.figshare.26131711.v1
WB CyTOF (raw)	This paper	Figshare: https://doi.org/10.6084/m9.figshare.26131711.v1
PBMC scRNAseq (pre-processed)	This paper	Figshare: https://doi.org/10.6084/m9.figshare.26131711.v1
PBMC scRNAseq (count tables and metadata)	This paper	GEO: GSE271284;

**Oligonucleotides**		

CpG2216	Miltenyi	130-100-243

**Software and algorithms**		

R	https://cran.r-project.org/	4.2.2 Patched
Ubuntu 20.04.5 LTS under WSL2, Windows 11	Microsoft, Canonical	N/A
Visual Studio Code	Microsoft	N/A
omiq.ai (cloud-based)	Dotmatics	N/A
Custom scripts for processing and analysis of CyTOF and scRNAseq data	This paper	Mendeley Data: https://doi.org/10.17632/kz7cpw3bnt.1
BatchAdjust (R script)	https://github.com/CUHIMSR/CytofBatchAdjust	used as inspiration for proprietary implementation
cytofkit (R package)	https://github.com/JinmiaoChenLab/cytofkit	0.99.0
uwot (R package)	https://github.com/jlmelville/uwot	0.1.14
ComplexHeatmap (R package)	http://bioconductor.org/packages/release/bioc/html/ComplexHeatmap.html	2.14.0
edgeR (R package)	https://bioconductor.org/packages/release/bioc/html/edgeR.html	3.40.1
ggplot2 (R package)	https://cran.r-project.org/web/packages/ggplot2/index.html	3.4.2
cowplot	https://cran.r-project.org/web/packages/cowplot/index.html	1.1.1
ggpubr	https://cran.r-project.org/web/packages/ggpubr/index.html	0.5.0
ggrepel	https://github.com/slowkow/ggrepel	0.9.2
ggfortify	https://cran.r-project.org/web/packages/ggfortify/index.html	0.4.15
flowCore	https://bioconductor.org/packages/release/bioc/html/flowCore.html	2.10.0
tidyverse	https://cran.r-project.org/web/packages/tidyverse/index.html	2.0.0
SeuratDisk	https://github.com/mojaveazure/seurat-disk	0.0.0.9020
Seurat	https://satijalab.org/seurat/	4.3.0
stringr	https://stringr.tidyverse.org/	1.5.0
DESeq2	https://bioconductor.org/packages/release/bioc/html/DESeq2.html	1.38.2
GSVA	https://bioconductor.org/packages/release/bioc/html/GSVA.html	1.46.0
enrichR	https://cran.r-project.org/web/packages/enrichR/vignettes/enrichR.html	3.1
fgsea	https://bioconductor.org/packages/release/bioc/html/fgsea.html	1.24.0
quasR	Doi: https://doi.org/10.1093/bioinformatics/btu781	N/A
pheatmap	Kolde, R. (2019). pheatmap: Pretty Heatmaps	N/A
class	https://cran.r-project.org/web/packages/class/index.html	7.3–22

**Other**		

High Sensitivity D5000 ScreenTape	Agilent	Cat# 5067-5592
Cytofix/Cytoperm Kit	BD	554714
Fixation Buffer	Biolegend	420801
True-Phos™ Perm Buffer	Biolegend	425401
Gibco™ HEPES (1 M)	gibco	15630–056
MEM Nicht essentielle Aminosäuren-Lösung (100×)	SIGMA-ALDRICH	M7145
Gibco™ GlutaMAX™ Supplement	gibco	35050–038
Natriumpyruvat (100 mM)	gibco	11360–039
Penicillin-Streptomycin	SIGMA-ALDRICH	P4333
CD45RA microbeads	Miltenyi	130-045-901
Invitrogen™ eBioscience™ CFSE	invitrogen	65-0850-84
eBioscience™ Cell Proliferation Dye eFluor™ 450	Thermo Fisher	88701
RPMI medium	gibco	31870–025
Pierce Universal Nuclease	Thermo Fisher	88702
FCS heat-inactivated	Sigma	S0615-500mL
Bovine Serum Albumin (BSA)	Miltenyi	130 091 376
PBS -	gibco	14190–094
EDTA	SIGMA-ALDRICH	03690
Beriglobin	CSL Behring	PZN 4616123
Pierce 16% Formaldehyde (w/v), Methanol-free	Thermo Fisher	Cat# 28908
Maxpar PBS	Standard BioTools	Cat# 201058
Maxpar Cell Staining buffer	Standard BioTools	Cat# 201068
Maxpar X8 Multimetal Labeling Kit	Standard BioTools	Cat# 201300
Nuclease-Free Water	Invitrogen	Cat# AM9937
TotalSeq-C0251 anti-human Hashtag 1	Biolegend	Cat# 394661; RRID: AB_2801031
TotalSeq-C0252 anti-human Hashtag 2	Biolegend	Cat# 394663; RRID: AB_2801032
TotalSeq-C0253 anti-human Hashtag 3	Biolegend	Cat# 394665; RRID: AB_2801033
TotalSeq-C0254 anti-human Hashtag 4	Biolegend	Cat# 394667; RRID: AB_2801034
TotalSeq-C0255 anti-human Hashtag 5	Biolegend	Cat# 394669; RRID: AB_2801035
TotalSeq-C0256 anti-human Hashtag 6	Biolegend	Cat# 394671; RRID: AB_2820042
TotalSeq-C0257 anti-human Hashtag 7	Biolegend	Cat# 394673; RRID: AB_2820043
TotalSeq-C0258 anti-human Hashtag 8	Biolegend	Cat# 394675; RRID: AB_2820044
TotalSeq-C0259 anti-human Hashtag 9	Biolegend	Cat# 394677; RRID: AB_2820045
TotalSeq-C0260 anti-human Hashtag 10	Biolegend	Cat# 394679; RRID: AB_2820046

### Experimental model and study participant details

#### Cohort 1/Berlin Pa-COVID-19 cohort

Pa-COVID-19 is a prospective observational cohort study assessing pathophysiology and clinical characteristics of patients with COVID-19 at Charité Universitätsmedizin Berlin.^19^ It is being carried out with the approval of the Institutional Review board of Charité (EA2/066/20). Written informed consent was provided by all patients or legal representatives for participation in the study. This cohort includes 50 (+8 patients with neutralizing IFN-I autoantibodies) COVID-19 infected adult patients. All COVID-19 patients were tested positive for SARS-CoV-2 RNA in nasopharyngeal swabs and allocated into mild (WHO 2–4) or severe (5–7) disease groups according to the WHO clinical ordinal scale. Please refer to Table S1 for a list of patient samples included in different experiments, including information about days post symptom onset, age and gender compositions of the cohort. Table S2 provides an overview of the most important cohort statistics.

#### Cohort 2/Berlin RECAST cohort

RECAST is a subproject of the Pa-COVID-19 observational study, aiming to characterize pathophysiology and SARS-CoV-2 infection progression primarily in patients under 18 years old.^5^ Data was collected from both minors and their family members in a longitudinal manner at three time points - directly after the diagnosis and at follow-up visits after approximately 2 weeks and 6 months. This cohort includes 40 SARS-CoV-2 infected-adults, 58 infected children, 35 control adult donors and 22 non-infected pediatric controls. None of the infected patients were hospitalized and thus fell under the WHO clinical ordinal scale of <3. Patients with either non-variant of concern (VOC) or Alpha (B.1.1.7) variant of SARS-CoV-2 were included in the analysis. Please refer to Table S1 for a list of patient samples included in different experiments, including information about days post symptom onset, age and gender compositions of the cohort. Table S2 provides an overview of the most important cohort statistics.

#### Age-matched elderly control cohort

Whole blood and PBMC samples of control donors over 50 (*n* = 14), that were included in CyTOF, scRNAseq and serum proteomics were obtained under protocols approved by the ethics committee of Charité – Universitätsmedizin Berlin (EA4/245/20 and EA4/244/20; EICOV and COVIMMUNIZE).^20^ All participants in the cohort provided written informed consent. Please refer to Table S1 for a list of patient samples included in different experiments, including information about days post symptom onset, age and gender compositions of the cohort. Table S2 provides an overview of the most important cohort statistics.

#### Control group definition

Control samples were defined as stemming from donors with a negative SARS-CoV-2 PCR test result without clinical signs of an ongoing infection.

### Method details

#### Antibodies used for mass cytometry (cohort 1 & 2)

All anti-human antibodies pre-conjugated to metal isotopes were obtained from Standard BioTools Corporation (San Francisco, USA). All remaining antibodies were obtained from the indicated companies as purified antibodies and in-house conjugation was done using the MaxPar X8 labeling kit (Standard BioTools, USA). Antibodies are listed in the key resources table.

#### Sample processing, antigen staining, and data analysis of mass cytometry-based immune cell profiling (cohort 1 & 2)

Sample processing, cell staining and acquisition was done as previously described.^17^

500μl of heparinized whole blood was stabilized by adding 700ul of proteomic stabilizer (Smart Tube Inc., San Carlos, US) following the manufacturer’s protocol and stored at −80°C until used. Blood samples were thawed using Thaw/Lyse buffer (Smart Tube Inc.). Antibodies targeting human beta-2 microglobulin (B2M) were conjugated in-house with metal isotopes 104Pd, 106Pd, 108Pd, 110Pd and 198Pt for barcoding. Up to 10 distinct samples were labeled simultaneously using Standard BioTools staining buffer with a pair of distinct B2M antibodies and incubated for 30 min at 4°C. After washing, cells were combined for subsequent surface and intracellular labeling. Cells were incubated in an antibody cocktail at 4°C for 30 min, washed with PBS and then fixed overnight with a 2% PFA solution prepared in PBS.

Intracellular staining involved washing cells twice in permeabilization buffer (eBioscience, San Diego, US), followed by incubation with intracellular antibodies diluted in permeabilization buffer at room temperature for 30 min. Cells were then washed and incubated with iridium intercalator solution (Standard BioTools) prepared in 2% PFA at room temperature for 20 min. Cells underwent an additional wash with PBS and two washes with ddH2O before storage at 4°C awaiting analysis by mass cytometry.

At minimum of 100,000 cells per sample was targeted and acquired using a Helios mass cytometer (Standard BioTools). For data normalization, EQ Calibration Beads (Standard BioTools) were added at a final dilution of 1:10. Immediately before acquisition, cells were resuspended in ddH2O, filtered using a 20-μm cell strainer (Celltrics, Sysmex), counted and diluted to achieve a concentration of 5-8x10^5^ cells/ml. Calibration beads at 1:10 v/v were included to correct for signal drift and daily sensitivity variations. Data were collected at a flow rate of 300–400 events per second. The lower convolution threshold was set at 400, noise reduction mode was activated and cells were defined using event duration ranging from 10 to 150 pushes. The resulting FCS files underwent normalization and randomization via CyTOF software using default software parameters.

OMIQ.ai cloud-based cytometry analysis software was used for de-barcoding of individual samples, manual gating of singlets and removal of normalization beads and dead cells. Per-channel intensity ranges were aligned between batches using a reference sample - a replicate acquired across all batches, and a proprietary script, based on BatchAdjust function, to compute scaling factors at the event percentiles of choice on per-channel basis.^66^ Populations of interest, such as T cells (defined as CD3^+^CD19^−^, HLA-DR^−/+^CD11c^−/+^, CD14^−^CD15^−^ cells and separated into CD4^+^TCRgd^−^ and CD8^+^TCRgd^−^ populations), B cells (defined as CD3^−^CD19^+^ and CD14^−^CD15^−^ cells) and monocyte-dendritic cell (DC) space (defined as CD3^−^CD19^−^, CD56^−^ and CD14^+^HLA-DR^−/+^ cells), were manually pre-gated (in an approach, similar to our previous project) and subset for further analysis in the R programming environment.^18^

The individual immune populations were then transformed using the inverse hyperbolic sine function (asinh) and *Z* score normalized per-marker across all samples and all events.

Datasets were clustered using FlowSOM algorithm as implemented in cytokfit R package (v. 0.99.0), setting the number of resulting clusters *k* as 30 (for CD4^+^ T cells) and 25 (for Monocyte-DC and B-cell datasets).^67^ A pre-selection of markers has been used as basis for clustering for each immune population subset (T cells: CD62L, CD45RO, CD27, CD28, CD226, ICOS, PD1, Lag3, TIGIT, CD96, CD25, CD38, CD56, CD69, CD137, HLADR, Ki67, CXCR3, CXCR5, CCR6, CRTH2, CD161, KLRG1, KLRF1, CD10, CD11c, CD123, CD16, CD95, CD34; B cells: IgD, IgM, CD10, CD21, HLADR, CXCR5, CD27, CD38, CD25, CXCR3, CD69, Ki67, CD95, CD11c, CD137, CCR6, CRTH2, CD62L, CD226, ICOS, PD1, Lag3, TIGIT, CD96, CD123, KLRG1, KLRF1, CD16, CD28, CD45RO, CD56, CD161, CD34; monocytes and DCs: CD14, CD16, HLADR, CD11c, CD123, CD8, CD10, CD69, CD38, CD62L, CD25, Ki67, CD226, CD95, CCR6, CRTH2, CXCR3, CXCR5, ICOS, PD1, Lag3, TIGIT, CD96, CD56, KLRG1, KLRF1, CD27, CD28, CD45RO, CD137, CD161, CD34).

The resulting clusters were then manually merged in a pairwise manner, based on their similarity in z-normalized marker expressions, to correct overclustering. For CD4^+^ T cells, measurement timepoints beyond acute infection were included (approximately two weeks and six months after the first visit). As these samples were added post-hoc, KNN classification algorithm was used to assign new cells to the existing cluster classes, using clustering of acute samples as training set (R package “class” v.7.3–22, function knn, default parameters). UMAPs were calculated on the same pre-selected markers, using the R package “uwot” (version 0.1.14, n_neighbors = 20, min_dist = 0.1, Euclidean distance).^68^ The frequency of each cluster was calculated as the percentage of cells in each cluster for each patient and for each immune cell compartment. Statistical testing for the difference in the frequency of each cluster across severity groups was calculated with the adjusted Wilcox test (Benjamini-Hochberg) for clusters with significant Kruskal-Wallis test (adjusted *p*-value (Benjamini-Hochberg) < 0.05, adjusted across all clusters in each immune cell compartment). For cluster abundance-age and average signal-age scatterplots, a linear model was fitted using “geom_smooth” function (ggplot2 package, version 3.4.0). For some analyses, activated T cell clusters were pre-selected, defined as clusters having above average z-scored expression of activation markers (CD25, HLA-DR, CD38, CD137, CD69, and Ki67). For scatterplots and boxplots showing per-patient average marker expression inside a population of interest, values have been *Z* score standardized inside the population of interest.

#### Isolation of blood cells for scRNA-seq (cohort 1 & 2)

Human peripheral blood mononuclear cells (PBMCs) were isolated from heparinized whole blood by density gradient centrifugation over Pancoll (density: 1,077g/ml, PAN-Biotech, Germany). Subsequently, the cells were counted, frozen and stored in liquid nitrogen. On the day of the experiment, the frozen PBMCs were thawed in pre-warmed thawing medium (RPMI 1640, Gibco; 2% FCS, Sigma; 0.01% Pierce Universal Nuclease, Thermo Fisher, USA).

#### 10x genomics chromium single-cell RNA-seq (cohort 1 & 2)

PBMCs were resuspended in staining buffer (DPBS, Gibco; 0.5% BSA, Miltenyi Biotec, Germany; 2 mM EDTA, Gibco, Thermo Fisher Scientific, USA) and hashtagged with 0.5 μg Total-Seq-C Hashtag antibodies for 30 min at 4°C. After the incubation, the PBMCs were washed three times, resuspended in DPBS, filtered through a 40 μm mesh (Flowmi Cell Strainer, Merck, Germany) and counted using the C-Chip hemocytometer (NanoEntek, South Korea). Subsequently, up to seven different samples were pooled equally.

The cell suspension was super-loaded with 40000–50000 cells per lane, in the Chromium Controller for partitioning single-cells into nanoliter-scale Gel Bead-In-Emulsions (GEMs).

In order to achieve a high enough cell number for each population on interest, the process above was repeated twice for each PBMC pool and additionally, B cells were enriched using untouched human B cell Isolation Kit II (Miltenyi Biotec, Germany) and loaded separately with approximately 20000 cells per lane.

The Chromium Next GEM Single Cell 5′ v.2 Dual Index kit was used for reverse transcription, cDNA amplification and library construction of the gene expression libraries (10x Genomics, USA). For additional VDJ and hashtag libraries the Chromium Single Cell V(D)J Enrichment Kit, Human T cell and Human B cell (10x Genomics, USA), as well as the Chromium Single Cell 5′ Feature Barcode Library Kit (10x Genomics, USA) were used. All libraries were prepared following the detailed protocols provided by 10x Genomics, quantified by Qubit Flex Fluorometer (Thermo Fisher, USA) and quality was checked using 4150 TapeStation automated electrophoresis system (Agilent, USA). Sequencing was performed in paired-end mode with an S1 and S2 flow cell using NovaSeq 6000 sequencer (Illumina, USA).

#### Human nasal swab scRNA-seq and pre-processed data treatment

Nasal swab data stems from a publication and is freely available.^8^ We, however, asked the authors to share a version of the dataset with non-normalized counts, suitable for pseudo bulk-based differential expression analysis. Please refer to the original publication for a detailed description of the experimental process, raw data pre-processing, quality control and integration.^8^

Integrated expression data were normalized by total UMI count per cell (log10(TP10k+1)) and scaled using Seurat (version 4.3.0) R package.^69^ Subsequently, immune cell populations of interest (T and plasmacytoid dendritic cells (pDCs)) were subset using cluster annotation from the original publication and treated separately. Principal component analysis (PCA) was performed on top 3000 variable genes and the first 15 PCs were used to construct a KNN graph and cluster the cells using Eucledian distance and Louvain algorithm (FindVariableFeatures, RunPCA, FindNeighbors and FindClusters functions, in that order) with the resolution of 0.5. This process has resulted in 14 and 8 clusters for T lymphocytes and pDCs, respectively. UMAP dimension reduction was also computed with the first 15 PCs in both cases using the default parameters. Further analysis was done using the same pipeline for both the nasal swab dataset and the PBMC dataset, please refer to the corresponding sections of the methods.

#### Pre-processing and integration of 10x genomics chromium PBMC scRNA-seq data (cohort 1 & 2)

Raw sequencing data were processed with CellRanger’s (v5) multi workflow and aligned against the GRCh38 reference, including TotalSeq C hashtag barcodes and VDJ data.

Cells from pooled samples were demultiplexed using a combination of HTODemux implemented in Seurat (v.4.3.0) and vireo (v0.5.6) after scoring common variants from the 1000Genomes project with cellsnp-lite (v1.2.0).^70^^,^^71^ Events classified as “Negative” and “Doublet” by the HTODemux algorithm were assigned an ID via vireo classification.

Demultiplexed batches of measurements were loaded into the environment and normalized separately, where gene expression values were normalized by total UMI counts per cell, multiplied by 10,000 (TP10K) and then log transformed by log10(TP10k+1) with NormalizeData Seurat function. Top 2000 variable features per batch were then selected and used to rank features for integration (FindVariableFeatures and SelectIntegrationFeatures functions). After per-batch scaling of gene expression and calculation of 50 PCs, integration was done using FindIntegrationAnchors and IntegrateData functions with reduction = “rpca” and dims = 1:50 parameters.

#### ScRNA-seq data analysis of 10x chromium dataset (cohort 1 & 2)

##### Data quality control

Subsequent to integration, cells were filtered by number of features (over 200 and less than 5000), percent mitochondrial genes (<10% mitochondrial UMIs) and number of counts per cell (<20000) to exclude debris and doublets.

#### Definition of the immune population spaces

Now integrated data were scaled and the first 15 of the newly computed PCs were used for clustering (resolution = 0.8) and UMAP calculation. Resulting 25 clusters were annotated based on their feature expression levels into non-granular subsets of main immune populations (T and B lymphocytes, NK cells, Monocytes, DCs) and subset into separate Seurat objects to be analyzed independently. Clusters that were either a mix of different immune cell lineages or did not have a high enough PTPRC gene expression were annotated as “drop” and excluded from further analysis.

#### Refining of separate immune populations and subclustering

To refine the data subsets, we filtered out cells, expressing genes exclusive to other populations. We made sure to clean T lymphocytes of gamma-delta T cells (defined as TRGC1>0 OR TRGC2>0); B lymphocytes of T cells, NK cells and monocytes (by removing CD3E > 0 OR CD3G > 0 OR CD3D > 0 OR NKG7>0 OR CD14 > 0 cells); Monocyte subset was refined through removing NK cells, B lymphocytes and T lymphocytes (NKG7>0 OR CD19 > 0 OR CD3E > 0 cells).

Following this, integrated data slot was used to rescale data and recalculate PCA values. The number of PCs to use for UMAP and clustering computation was selected by analyzing an elbow plot and it varied for different subsets: 15 for T cells, 14 for B cells, 15 for DCs and 17 for monocytes. NK cells were not analyzed, as mass cytometry did not show many clear patterns in this immune space.

Subclustering was calculated with a resolution of 1 for T cells and 0.5 for all other subsets. UMAP was calculated with default parameters for all subsets. Same as previously, scaling, PCA, UMAP and clustering calculation was done using Seurat R package (v.4.3.0).

#### Cluster annotation and statistical testing

Subclustering of separate immune populations has netted 21 clusters (of which 2 were dropped) for T cells, 13 clusters (of which 1 was dropped) for B cells, 11 clusters (of which 1 was dropped) for Monocytes and 11 clusters (of which 1 was dropped) for DCs. Annotation of clusters has been done using a combination of manually pre-selected marker genes and automatically detected cluster-specific genes (using FindAllMarkers function, showing top 10 significantly enriched genes, detected in at least 25% of events and having a log-fold-change of at least 0.25). Manual labels were assigned to each cluster. In some cases, if clusters were highly similar, the same label was assigned, merging the cluster for further analysis.

Annotated cluster abundances were compared between the respective groups and the statistical significance was calculated using the adjusted Wilcox test (Benjamini-Hochberg) for clusters with significant Kruskal-Wallis test (adjusted *p*-value (Benjamini-Hochberg) < 0.05, adjusted across all annotated clusters in each immune cell compartment). Adult patients with anti-IFN-a autoantibodies were removed from infected adults - infected children comparison to keep the comparison more representative of the general population.

Analysis of cluster frequencies has allowed us to pre-select annotated clusters expanded in acute SARS-CoV-2 infection, thus making further analysis more informative through isolation of infection-specific effects. If not specifically stated otherwise, differential gene expression (DE) analysis and analyses based on its output were done using cells from expanded clusters.

For T lymphocyte space, CD8^+^ T lymphocytes were defined as CD8A AND CD8B expressing cells, with CD4^+^ identity being assigned to the rest of the cells.

#### Differential expression (DE) and gene ontology (GO) enrichment analysis

For the identification of differentially expressed genes between disease groups, we used pseudobulk gene expression, defined as the sum of the raw counts from all cells of each patient among selected clusters of interest. The pseudobulk samples were then normalized and modeled according to the DESeq2 pipeline (v. 1.38.2).^72^ Normalization was done using DESeq2’s median-of-ratios method, the DESeq2 function DESeq() was used to estimate size factors, dispersion, and to fit a generalized linear model for each gene. Patient group was included as a sole factor. Differential expression was assessed using the Wald test for each gene. *p*-values were adjusted for multiple testing using the Benjamini-Hochberg method to control the false discovery rate (FDR).

For further enrichment analysis, we selected differentially expressed genes with high enough counts (“baseMean” > 50) and *p*-value lower than 0.05. GO enrichment analysis was performed with the R package “enrichR” (v.3.1) and "GO_Biological_Process_2018" database.^73^

#### Gene set enrichment analysis (GSEA)

The log2-fold change of differentially expressed genes from DESeq2 was used to define the ranked gene list used for GSEA. We tested different annotated gene lists for different immune populations (Table S3). GSEA was performed with the R package “fgsea” (v. 1.24.0) with 1000 permutations for statistical testing.^74^

#### Clonal composition analysis

Our experimental design included VDJ region sequencing to enable clonal composition analysis. We used an R package called scRepertoire (v. 1.8.0) to do that.^75^ scRepertouire interacts with the Seurat object to combine gene expression data and clonotype information that can be called from the VDJC gene or the sequence of the CD3R region. In our case we mostly use the “strict” option for calling the clonotype, which uses both the gene and the nucleotide. Clonal overlap proportions between plasmablasts and other expanded clusters have been calculated via “clonalNetwork” function and used as the basis for the stacked barchart plot, with the clone calling method being set to “gene” in order to increase sensitivity.

#### Data visualization

All the graphical visualization of the data was performed in R with the ggplot2 package apart from the heatmaps, which were displayed using the ComplexHeatmap (v. 2.14.0) and pheatmap (v. 1.0.12) packages.^76^

Boxplots:

Boxplots are calculated in the style of Tukey, shortly, the center of the box represents the median of the values, the hinges the 25th and 75th percentile and the whiskers are extended no further than the 1.5 ∗ IQR (interquartile range).

#### Heatmap

The heatmap shows the mean value of the z-scaled expression of each marker (or abundance, cytokine concentration) in each cluster or patient group.

#### Dot plot

The dot plot of the signature genes shown was calculated according to the Dotplot Seurat function scaling the expression values by gene.

#### Detection of SARS-CoV-2-specific IgG and IgA antibodies

For the detection of IgG and IgA to the S1 domain of the SARS-COV-2 spike (S) protein, anti-SARS-CoV-2 assay was used according to the manufacturer's instructions (Euroimmun, Lübeck, Germany). Serum samples were tested at a 1:101 dilution using the fully EUROIMMUN Analyzer. Optical density (OD) ratios were calculated by dividing the OD at 450 nm by the OD of the calibrator included in the kit. The calculated OD ratios can be used as a relative measure for the concentration of antibodies in the serum.^77^

#### *Ex vivo* functional analyses of T cells

##### Cell purification

Frozen PBMCs of control donors were thawed and washed with a Benzonase-containing medium (RPMI, 2% FCS, Pierce Universal Nuclease, 250U/mL), transferred to MACS buffer (PBS, 0.5% BSA, 2mM EDTA) and counted. Cells were split into CD45RA^+^ and CD45RA^−^ fractions using magnetic separation with CD45RA MicroBeads (Miltenyi Biotec) according to the manufacturer`s protocol. CD45RA-fraction was labeled with eF450 proliferation dye in PBS- for 10 min at room temperature (10μM final eF450 concentration). Labeling was stopped by adding 4 volumes of cold complete medium (containing 10% FCS) and cells were incubated on ice for 5 min followed by two washing steps with RPMI/5%FCS. CD45RA^+^ fraction was labeled with CFSE proliferation dye in RPMI for 7 min at room temperature (5uM final CFSE concentration) and washed thrice with RPMI/5%FCS, after which cells rested on ice for 30 min. Labeled cells were then pooled for each donor and volume was adjusted to 2000 cells/ul in complete medium (filtrated RPMI +glutamine, 10% (V/V) heat inactivated FCS, 1% HEPES, 1% NEAA, 1% GlutaMAX, 1% sodium pyruvate, 1% Pen. Strep.).

#### Stimulation approach

A 96-well, rounded bottom plate was used for the culture, to accommodate all the challenge conditions and donor groups per batch. If the cell number was high enough, two replicates of each condition per donor were included. Reagents were diluted in 100ul of complete medium to a final concentration of 0.324μM ODN CpG2216, 100ng/ml SEB, 0.5ng/ml or 30ng/ml recombinant IFNa, 1ug/ml B18R, 20mg/ml recombinant IL-1b and 10 μg/ml Anakinra (“Kineret”) in following combinations: SEB, SEB+CpG2216, SEB+CpG2216 + B18R, SEB+CpG2216+IL-1b, SEB+CpG2216+Anakinra, SEB+rec. IFNa and SEB+rec. IFNa+IL-1b and placed into respective wells. Following this, 100ul of cell suspension per well have been mixed into the reagent containing medium, thus achieving the equal amount of 200000 cells per well. Cells were then cultured for 96 h at 37°C and 5% CO2.

#### Full-spectrum flow cytometry measurement and analysis

After 96 h, cell culture supernatant was harvested and frozen at −80°C. Cells were washed with MACS buffer and resuspended in either 50ul (for pooled duplicates) or 30ul (for single wells) of freshly prepared surface staining antibody mix, including a live-dead marker (see key resources table) and incubated for 30 min at 4°C in the dark. Marker panel has been designed, based on the results detected in CyTOF and scRNAseq experiments. Subsequently, cells were washed twice with cold MACS buffer and fixed in either 100ul Cytofix/Cytoperm buffer (BD Biosciences, USA, for pooled duplicates) or 50ul Cytofix/Cytoperm buffer (for single wells). After a 20 min incubation at 4°C in the dark, cells were washed twice with Cytoperm wash buffer and resuspended in freshly prepared intracellular staining antibody mix (antibodies were diluted in Cytoperm wash buffer to keep cells permeabilized). Cells were incubated for 30 min at 4°C in the dark, washed once with Cytoperm wash buffer, once with MACS buffer, transferred to plastic FACS tubes, resuspended in 150ul of MACS buffer and immediately measured. Cytek Aurora (Cytek Biosciences, USA) full-spectrum flow cytometer was used to acquire all the samples. SpectroFlo (Cytek Biosciences, USA) software was used for spectral unmixing (compensation) of fluorophore signals using SEB+CpG2216-activated unstained reference samples of adult donors, measured with every batch. Single-stained references were prepared either with compensation beads (CompBeads, BD Biosciences, USA) or SEB+CpG2216-activated adult donors` cells, depending on the marker expression level and fluorophore intensity. Compensated data was uploaded to OMIQ.ai cloud-based cytometry analysis software for gating and export of population abundances and median signal intensities. Exported data was imported into R programming environment, where it was aggregated and analyzed.

#### Quantification of cytokines, chemokines

Plates with frozen cell culture supernatants were thawed on ice and centrifuged at 1000xg at 4°C for 10 min to remove particulates. Cytokine/Chemokine/Growth Factor 45-Plex Human ProcartaPlex Panel 1 kit (ThermoFischer Scientific, USA) and a custom 18-Plex Human ProcartaPlex Mix&Match panel (ThermoFischer Scientific, USA) were used in combination with Luminex xMAP technology-based Bio-Plex 200 system (Bio-Rad Laboratories, Inc., USA) according to manufacturer`s protocols. Resulting dataset was imported into R programming environment for analysis. Custom panel specifics can be found in the resources table.

#### Blood serum cytokine and chemokine quantification

Blood serum samples of patients and control donors have been measured using CorPlex Human Cytokine 10-plex panel 1 assay (Quanterix Corp., USA) on Simoa SP-X (Quanterix Corp., USA) system and IFN-a assay (Quanterix Corp., USA) on Simoa HD-X (Quanterix Corp., USA) system, respectively. Serum samples were thawed on ice and centrifuged at 10000xg for 5 min at 4°C to remove particulates and clarify the sample, after which the assays were performed following the detailed protocols provided by the manufacturer. Resulting data has been imported into R programming environment, where it was aggregated and analyzed.

#### *Ex vivo* STAT1 and STAT3 phosphorylation assay

##### Cell purification

Frozen PBMCs of control donors were thawed and washed with a Benzonase-containing medium (RPMI, 2% FCS, Pierce Universal Nuclease, 250U/mL). Volume was adjusted for each donor sample to achieve the concentration of 2000 cells/ul in complete medium (filtrated RPMI +glutamine, 10% (V/V) heat inactivated FCS, 1% Pen. Strep.).

#### Stimulation approach

A 96-well, rounded bottom plate was used for the culture, to accommodate all the challenge conditions and donor groups per batch. If the cell number was high enough, two replicates of each condition per donor were included. Reagents were diluted in 100ul of complete medium to a final concentration of 100ng/ml SEB and 30ng/ml recombinant IFNa in following combinations: SEB, SEB+rec. IFNa and placed into respective wells. Following this, 100ul of cell suspension per well have been mixed into the reagent containing medium, thus achieving the equal amount of 200000 cells per well. Cells were then incubated for 25 min at 37°C and 5% CO2.

10 min before the end of the incubation period, a live-dead staining agent was added (eF506, at 1:100 dilution).

#### Flow cytometry measurement and analysis

After 25 min of incubation, duplicate wells were pooled in total volume of 200ul, washed in FACS buffer (two rounds, 300xg, 5 min, at room temperature) and immediately fixated using pre-warmed (37°C) commercially available fixation buffer, according to manufacturer`s protocol (BioLegend Cat 420801). After 15 min of fixation at 37°C, cells were washed in FACS buffer (2 times, 500xg, 5 min, at room temperature) and subjected to Beriglobin blocking (in 25ul for duplicate wells, 20 min at 4°C, 160mg/ml stock, used in 1:50). After a single wash step (500xg, 5 min, RT), cells were resuspended in 50ul (for pooled wells) of freshly prepared surface staining solution and incubated for 30 min at 4°C in the dark n (see key resources table for details on cytometry panels). Two washing steps (FACS buffer, 500xg, 5 min, RT) were followed by permeabilization using 200ul True-Phos perm buffer per well, pre-chilled to −20°C according to the manufacturer`s protocol (BioLegend Cat 425401). After the required 1 h incubation at −20°C in the dark, permeabilized cells were washed twice in FACS buffer (1000xg, 5 min, RT), resuspended in 100ul of freshly prepared intracellular staining solution (see key resources table for details on cytometry panels) and incubated for 30 min in the dark at room temperature. Cells were subsequently washed twice (FACS buffer, 1000xg, 5 min, RT), resuspended in 100ul FACS buffer per well and stored at 4°C in the dark until measurement next day.

BD FACSymphony A5 Cell Analyzer was (BD Biosciences, USA) used to acquire all the samples. FACSDiva software was used for acquisition and compensation of samples. Single-stained references were prepared either with compensation beads (CompBeads, BD Biosciences, USA) or SEB+IFNa-activated adult donors` cells, depending on the marker expression level and fluorophore intensity.

Compensated data was uploaded to OMIQ.ai cloud-based cytometry analysis software for gating and export of population abundances and median signal intensities. Exported data was imported into R programming environment, where it was aggregated and analyzed.

#### Bulk RNAseq of whole blood of SARS-CoV-2-infected hamsters

Details about the animal experiment itself can be found in the original publication.^37^ As stated in the original publication, the animal experiments were approved by the Landesamt für Gesundheit und Soziales in Berlin, Germany (approval number 0086/20).^37^

To extract RNA from whole hamster blood, previously frozen samples were diluted 1:1 with PBS, added to TrizolLS (Invitrogen) reagent at the manufacturer recommended proportion of 1:3 (sample:TrizolLS) and vortexed. After 20min of inactivation, samples were transferred from the BSL3 to a BSL2 facility. RNA extraction was continued according to the manufacturers protocol. RNA was eluted in RNAse free water and stored at −80°C until sequencing.

For RNA extraction from hamster tissue, 50–100 mg of lung tissue was homogenized using a bead mill (Analytic Jena) for 30s, then 1mL of Trizol reagent (Invitrogen) was added to the tubes. After an additional homogenisation step (30s) samples were transferred to fresh tubes and incubated for 20min to ensure inactivation before moving them to the BSL2 facility. RNA was then extracted according to the manufacturers instructions, eluted in RNAse free water and stored at −80°C.

Sequencing libraries were generated using the the QuantSeq 3′ mRNA-Seq V2 Library Prep Kit FWD (Lexogen, cat# 193.384) according the manufacturer’s instruction, and sequenced on a Novaseq X device (Illumina) on a 25B flowcell to a depth of about 20–30 million reads per sample. Read 1 of length 151 nucleotides was aligned to the MesAur 2.0 genome assembly (https://www.ncbi.nlm.nih.gov/genome/11998?genome_assembly_id=1585474). The GTF file used for gene counting was described previously.^78^ For data analysis, the R packages quasR, dplyr and and pheatmap (Kolde, R. (2019). pheatmap: Pretty Heatmaps) were used.^79^

All work involving live SARS-CoV-2 virus was conducted under appropriate biosafety conditions in the BSL-3 facility at Institut für Virologie, Freie Universität Berlin, Germany. All animal experimentation was approved by the competent state authority (Landesamt für Gesundheit und Soziales in Berlin, Germany, permit number 0086/20) and performed in compliance with all relevant national and international guidelines for care and humane use of animals.

### Quantification and statistical analysis

The software used for statistical analysis is stated in the paragraphs describing the respective experimental procedures in the section “methods details.” For statistical details please refer to the figure legends and the respective experimental procedures in the section “methods details.”

The study was not blinded, and the sample size was calculated empirically, prioritizing the inclusion of the highest number of COVID-19 samples and matching controls.

One ”n” represents the results of one experiment conducted with specimens of one individual at one time point. In the case of sequential analysis i.e., visit 1 versus visit 2, it refers to specimens from identical donors at two different time points.

If no absolute *p* value is shown in the comparison: ∗, *p* < 0.05; ∗∗, *p* < 0.01; ∗∗∗, *p* < 0.001; ∗∗∗∗, *p* < 0.0001.

#### Additional resources

Pa-COVID-19 clinical trial registered with German Clinical Trials Register: DRKS00021688.

RECAST clinical trial registered with German Clinical Trials Register: DRKS00025715.

EICOV/COVIMMUNIZE (COVIM) clinical trial registered with EU Clinical Trials Register: EudraCT number 2021-001512-28.

## Supplemental information

Document S1. Figures S1–S7 and Tables S2 and S3

Table S1. Detailed metadata of patient samples used in experiments, related to all Figures (A) Metadata of IFNa autoantibody-negative patient samples. (B) Metadata of IFNa autoantibody-positive patient samples. (C) Detailed stratification information for each experiment and figure presented.

Table S4. Gene sets, used for scRNA-seq cluster annotation and GSEA (A) Manually curated list of genes for annotation of immune populations. (B) Top 10 unique enriched genes for each marker, resulting from FindAllMarkers Seurat function applied to monocyte population from PBMC dataset. (C) Top 10 unique enriched genes for each marker, resulting from FindAllMarkers Seurat function applied to T cell population from PBMC dataset. (D) Top 10 unique enriched genes for each marker, resulting from FindAllMarkers Seurat function applied to B cell population from PBMC dataset. (E) Top 10 unique enriched genes for each marker, resulting from FindAllMarkers Seurat function applied to T cell population from nasal swab dataset. (F) Gene sets, used for GSEA.

Document S2. Article plus supplemental information
