# Supplementary material for: Rewired type I IFN signaling is linked to age-dependent differences in COVID-19
Source: Cell Rep Med. 2025 Aug 19;6(8):102285. doi: 10.1016/j.xcrm.2025.102285 (PMC12432374; doi:10.1016/j.xcrm.2025.102285)
Supplement: Document S1. Figures S1–S7 and Tables S2 and S3 [file mmc1.pdf]

Cell Reports Medicine, Volume *6*

Supplemental information

Rewired type I IFN signaling is linked to age-dependent differences in COVID-19

Lev Petrov, Sophia Brumhard, Sebastian Wisniewski, Philipp Georg, David Hillus, Anna Hiller, Rosario Astaburuaga-García, Nils Blüthgen, Emanuel Wyler, Katrin Vogt, Hannah-Philine Dey, Saskia von Stillfried, Christina Iwert, Roman D. Bülow, Bruno Märkl, Lukas Maas, Christine Langner, Tim Meyer, Jennifer Loske, Roland Eils, Irina Lehmann, Benjamin Ondruschka, Markus Ralser, Jakob Trimpert, Peter Boor, Sammy Bedoui, Christian Meisel, Marcus A. Mall, Victor M. Corman, Leif Erik Sander, Jobst Röhmel, and Birgit Sawitzki

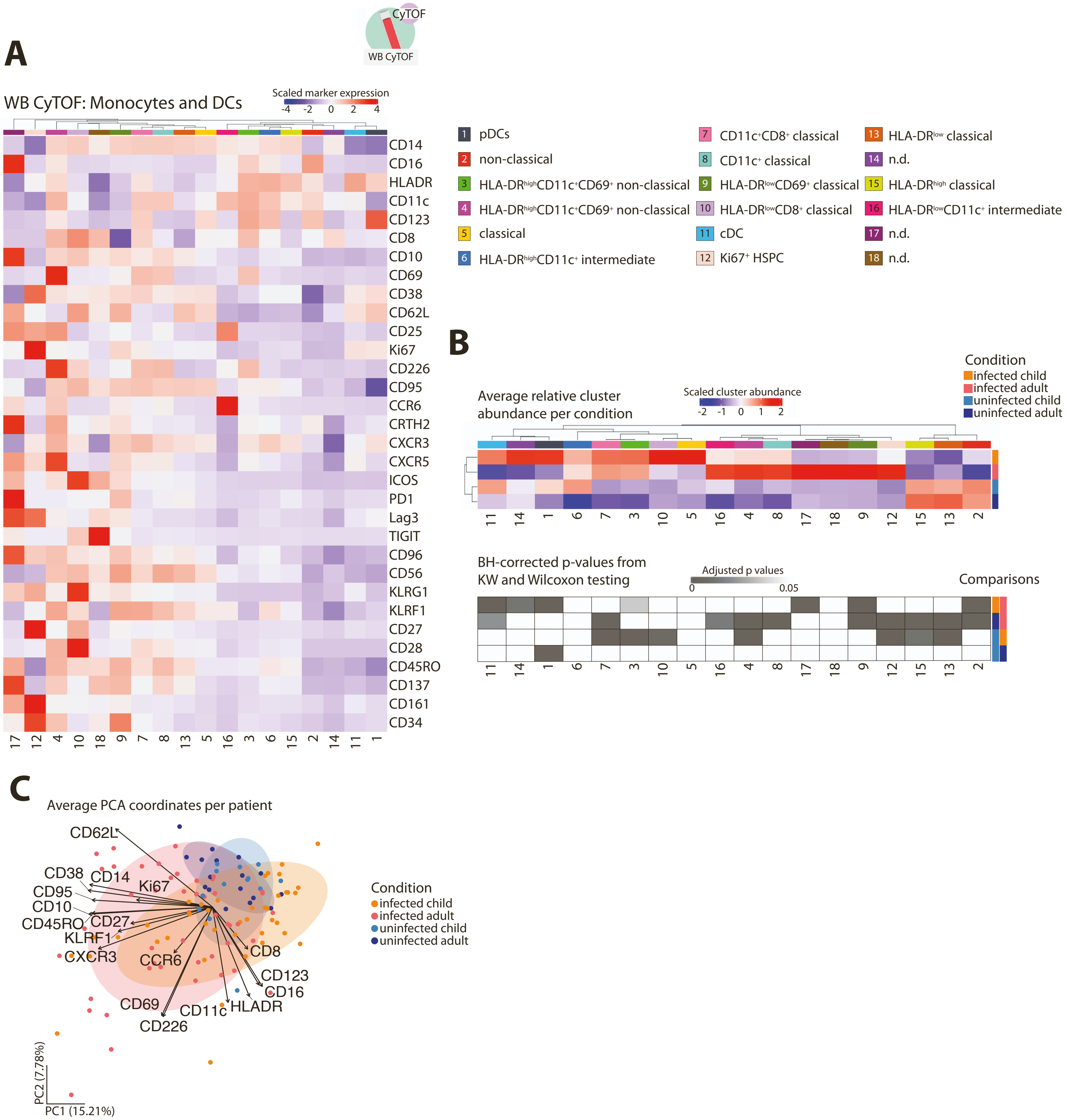

CyTOF
WB CyTOF
A
WB CyTOF: Monocytes and DCs
Scaled marker expression
-4 -2 0 2 4
CD14
CD16
HLADR
CD11c
CD123
CD8
CD10
CD69
CD38
CD62L
CD25
Ki67
CD226
CD95
CCR6
CRTH2
CXCR3
CXCR5
ICOS
PD1
Lag3
TIGIT
CD96
CD56
KLRG1
KLRF1
CD27
CD28
CD45RO
CD137
CD161
CD34
17 12 4 10 18 9 7 8 13 5 16 3 6 15 2 14 11 1
1 pDCs
2 non-classical
3 HLA-DRhighCD11c+CD69+ non-classical
4 HLA-DRhighCD11c+CD69+ non-classical
5 classical
6 HLA-DRhighCD11c+ intermediate
7 CD11c+CD8+ classical
8 CD11c+ classical
9 HLA-DRlowCD69+ classical
10 HLA-DRlowCD8+ classical
11 cDC
12 Ki67+ HSPC
13 HLA-DRlow classical
14 n.d.
15 HLA-DRhigh classical
16 HLA-DRlowCD11c+ intermediate
17 n.d.
18 n.d.
B
Average relative cluster abundance per condition
Scaled cluster abundance
-2 -1 0 1 2
Condition
infected child
infected adult
uninfected child
uninfected adult
11 14 1 6 7 3 10 5 16 4 8 17 18 9 12 15 13 2
BH-corrected p-values from KW and Wilcoxon testing
Adjusted p values
0 0.05
Comparisons
11 14 1 6 7 3 10 5 16 4 8 17 18 9 12 15 13 2
C
Average PCA coordinates per patient
CD62L
CD38
CD14
Ki67
CD95
CD10
CD45RO
CD27
KLRF1
CXCR3
CCR6
CD8
CD123
CD16
CD69
CD11c
HLADR
CD226
PC2 (7.78%)
PC1 (15.21%)
Condition
infected child
infected adult
uninfected child
uninfected adult

Figure S1. Annotation and statistical analysis of monocyte clusters identified by CyTOF, related to Figure 1

(A) Heatmap, containing z-score normalized average expression values of each monocyte & dendritic cell cluster resulting from a semi-supervised FlowSOM clustering approach of CyTOF data from whole blood samples (all the patients included in the dataset and used for clustering: uninfected children (RECAST n = 13; median age = 7), uninfected adults (RECAST n = 25; median age = 48), infected children (RECAST study n = 48; median age = 8; asymptomatic n = 11, symptomatic n = 37) and adults (RECAST n = 21, PA-COVID study n = 30; median age = 47; mild n = 37, severe n = 10, severe with IFN autoantibodies n = 4). The dendrogram in the upper part of the panel shows the result of distance-based hierarchical clustering. A color-coded annotation of each cluster is presented on the right, n.d. = not defined.

(B) Heatmap, showing scaled mean cluster abundance of monocyte and DC clusters for each patient group (above) and a heatmap presenting the results of statistical testing for all relevant comparisons (below; Benjamini-Hochberg-corrected p values from non-parametric Kruskal-Wallis with post-hoc Wilcoxon). Severe patients with IFN autoantibodies were excluded from the analysis.

(C) PCA plot, presenting average principal component 1 and 2 coordinates of all monocyte and DC cells of each patient (colored according to patient group). The plot contains arrows labeled with marker names. These arrows represent the importance of each marker for the calculation of the components. The direction shows which principal components the marker correlates with, and the length of the arrow illustrates the magnitude of the variable's contribution to the principal components. Each arrow points in the direction where the variable it represents increases in the principal component space and longer arrows indicate that the variable has a strong influence on the principal components. Severe patients with IFN autoantibodies were excluded from the analysis.

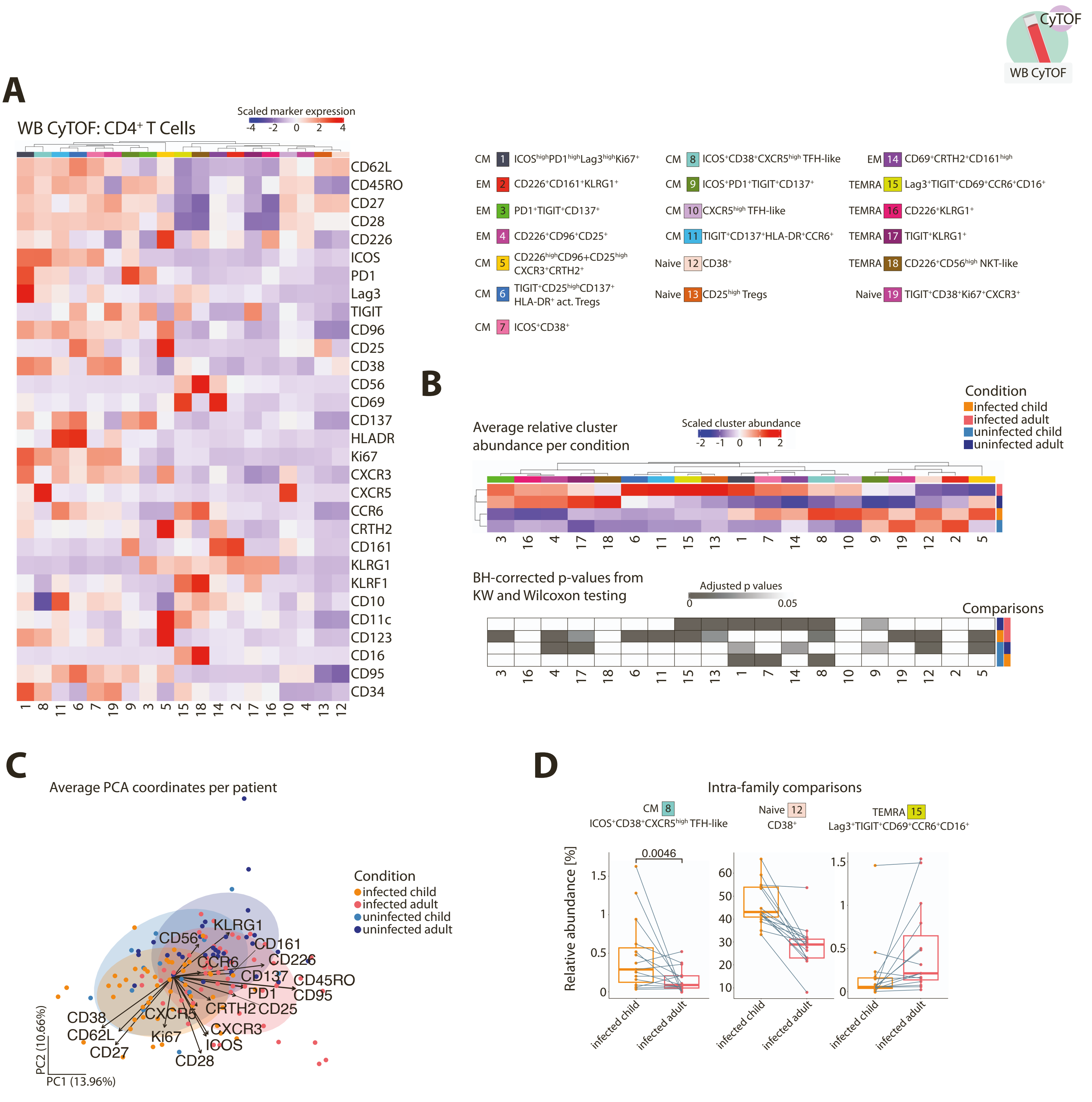

CyTOF
WB CyTOF
A
WB CyTOF: CD4+ T Cells
Scaled marker expression
-4 -2 0 2 4
CD62L
CD45RO
CD27
CD28
CD226
ICOS
PD1
Lag3
TIGIT
CD96
CD25
CD38
CD56
CD69
CD137
HLADR
Ki67
CXCR3
CXCR5
CCR6
CRTH2
CD161
KLRG1
KLRF1
CD10
CD11c
CD123
CD16
CD95
CD34
1 8 11 6 7 19 9 3 5 15 18 14 2 17 16 10 4 13 12
CM 1 ICOS^high PD1^high Lag3^high Ki67+
EM 2 CD226+CD161+KLRG1+
EM 3 PD1+TIGIT+CD137+
EM 4 CD226+CD96+CD25+
CM 5 CD226^high CD96+CD25^high CXCR3+CRTH2+
CM 6 TIGIT+CD25^high CD137+ HLA-DR+ act. Tregs
CM 7 ICOS+CD38+
CM 8 ICOS+CD38+CXCR5^high TFH-like
CM 9 ICOS+PD1+TIGIT+CD137+
CM 10 CXCR5^high TFH-like
CM 11 TIGIT+CD137+HLA-DR+CCR6+
Naive 12 CD38+
Naive 13 CD25^high Tregs
EM 14 CD69+CRTH2+CD161^high
TEMRA 15 Lag3+TIGIT+CD69+CCR6+CD16+
TEMRA 16 CD226+KLRG1+
TEMRA 17 TIGIT+KLRG1+
TEMRA 18 CD226+CD56^high NKT-like
Naive 19 TIGIT+CD38+Ki67+CXCR3+
B
Average relative cluster abundance per condition
Scaled cluster abundance
-2 -1 0 1 2
Condition
infected child
infected adult
uninfected child
uninfected adult
3 16 4 17 18 6 11 15 13 1 7 14 8 10 9 19 12 2 5
BH-corrected p-values from KW and Wilcoxon testing
Adjusted p values
0 0.05
Comparisons
3 16 4 17 18 6 11 15 13 1 7 14 8 10 9 19 12 2 5
C
Average PCA coordinates per patient
Condition
infected child
infected adult
uninfected child
uninfected adult
KLRG1
CD56
CD161
CD226
CCR6
CD137
CD45RO
PD1
CD95
CRTH2
CD25
CXCR5
CD38
CD62L
Ki67
CXCR3
ICOS
CD27
CD28
PC2 (10.66%)
PC1 (13.96%)
D
Intra-family comparisons
CM 8
ICOS+CD38+CXCR5^high TFH-like
Naive 12
CD38+
TEMRA 15
Lag3+TIGIT+CD69+CCR6+CD16+
Relative abundance [%]
0.0046
1.5
1
0.5
0
60
50
40
30
20
10
1.5
1
0.5
0
infected child
infected adult
infected child
infected adult
infected child
infected adult

Figure S2. Annotation and statistical analysis of $CD4^+$ T-cell clusters identified by CyTOF, related to Figure 2

(A) Heatmap, containing z-score normalized average expression values of each $CD4^+$ T-cell cluster resulting from a semi-supervised FlowSOM clustering approach of CyTOF data from whole blood samples (all the patients present in the dataset and used for clustering including follow-up measurements of some patients done approximately two weeks and six months after the first, acute infection phase measurement, were included for the heatmap calculation). Patient groups shown are: uninfected children (RECAST n = 13; median age = 7), uninfected adults (RECAST n = 25; median age = 48), infected children (RECAST study n = 53; median age = 8; asymptomatic n = 11, symptomatic n = 42) and adults (RECAST n = 24, PA-COVID study n = 51; median age = 51; mild n = 45, severe n = 24, severe with IFN autoantibodies n = 6). The dendrogram in the upper part of the panel shows the result of distance-based hierarchical clustering. A color-coded annotation of each cluster is presented on the right.

(B) Heatmap, showing scaled mean cluster abundance of $CD4^+$ T-cell clusters for each patient group (above) and a heatmap presenting the results of statistical testing for all relevant comparisons (below; Benjamini-Hochberg-corrected p values from non-parametric Kruskal-Wallis with post-hoc Wilcoxon). Importantly, only samples measured during the acute phase of infection (defined as first 14 days after symptom onset) were used for calculation. Severe patients with IFN autoantibodies were excluded from the analysis. Group composition is thus as follows: uninfected children (RECAST n = 13; median age = 7), uninfected adults (RECAST n = 25; median age = 48), infected children (RECAST study n = 48; median age = 8; asymptomatic n = 11, symptomatic n = 37) and adults (RECAST n = 21, PA-COVID study n = 26; median age = 45; mild n = 37, severe n = 10).

(C) PCA plot, presenting average principal component 1 and 2 coordinates of all $CD4^+$ T cells of each patient (colored according to patient group). The plot contains arrows labeled with marker names. These arrows represent the importance of each marker for the calculation of the components. The direction shows which principal components the marker correlates with and the length of the arrow illustrates the magnitude of the variable's contribution to the principal components. Simply put, each arrow points in the direction where the variable it represents increases in the principal component space and longer arrows indicate that the variable has a strong influence on the principal components. Severe patients with IFN autoantibodies were excluded from the analysis.

(D) Box plots, showing relative frequencies of annotated clusters, resulting from the FlowSOM algorithm, applied to the $CD4^+$ T-cell CyTOF data. Cluster abundance is compared between infected children (RECAST study n = 20; median age = 9; asymptomatic n = 3, symptomatic n = 17) and infected adults (RECAST study n = 19; median age = 39; mild n = 19) within the same family. Abundances were averaged, if multiple parents (mother and father) or children were measured in a family (family n = 15). Lines connect parents and children from the same family. Only samples measured during the acute phase of infection (defined as first 14 days after symptom onset) are shown. Statistical testing was done using pairwise Wilcoxon rank sum test.

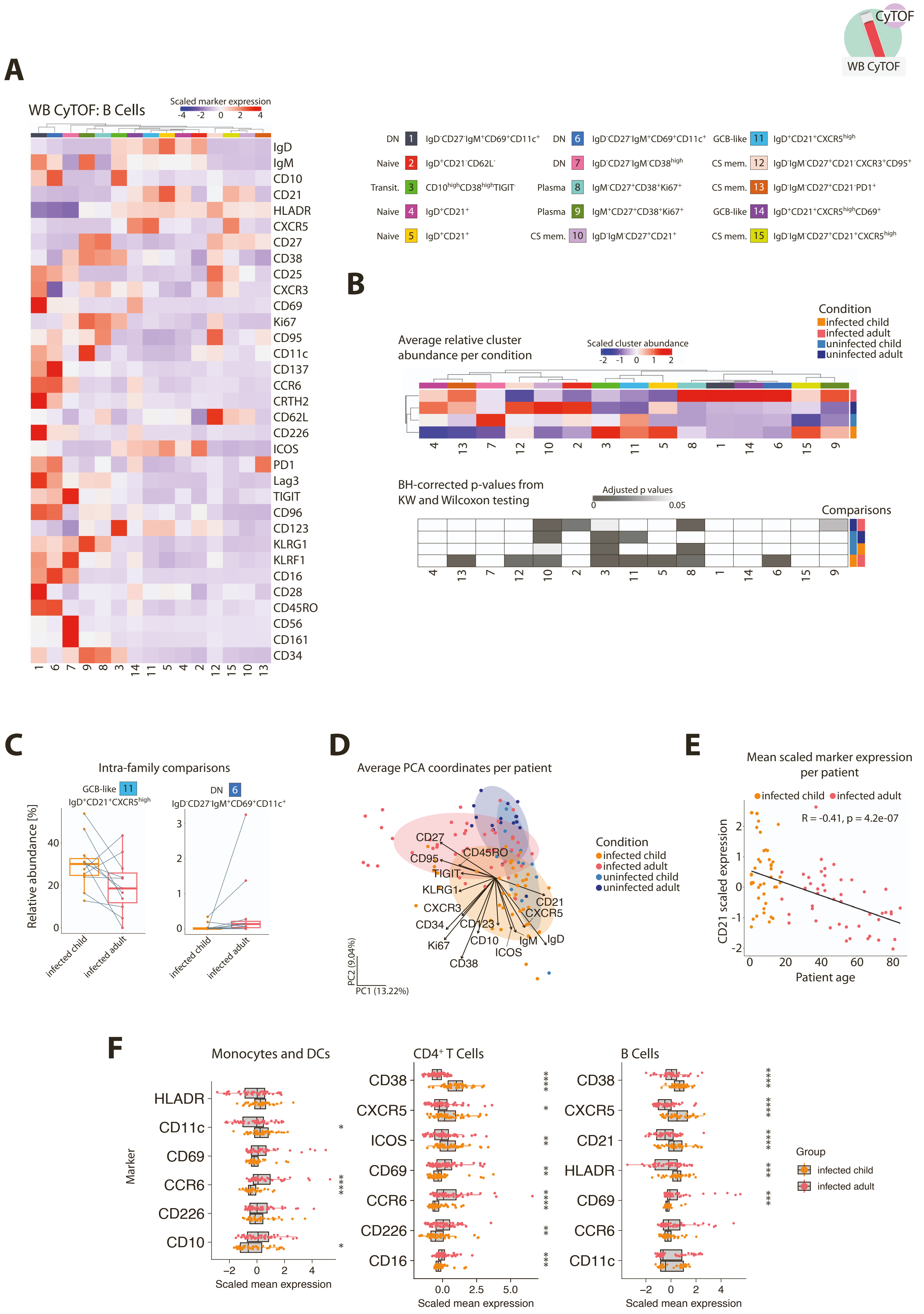
CyTOF
WB CyTOF
A
WB CyTOF: B Cells
Scaled marker expression
IgD
IgM
CD10
CD21
HLADR
CXCR5
CD27
CD38
CD25
CXCR3
CD69
Ki67
CD95
CD11c
CD137
CCR6
CRTH2
CD62L
CD226
ICOS
PD1
Lag3
TIGIT
CD96
CD123
KLRG1
KLRF1
CD16
CD28
CD45RO
CD56
CD161
CD34
DN 1 IgD-CD27-IgM+CD69+CD11c+
Naive 2 IgD+CD21-CD62L-
Transit. 3 CD10highCD38highTIGIT-
Naive 4 IgD+CD21+
Naive 5 IgD+CD21+
DN 6 IgD-CD27-IgM+CD69+CD11c+
DN 7 IgD-CD27-IgM-CD38high
Plasma 8 IgM-CD27+CD38+Ki67+
Plasma 9 IgM+CD27+CD38+Ki67+
CS mem. 10 IgD-IgM-CD27+CD21+
GCB-like 11 IgD+CD21+CXCR5high
CS mem. 12 IgD-IgM-CD27+CD21-CXCR3+CD95+
CS mem. 13 IgD-IgM-CD27+CD21-PD1+
GCB-like 14 IgD+CD21+CXCR5highCD69+
CS mem. 15 IgD-IgM-CD27+CD21+CXCR5high
B
Average relative cluster abundance per condition
Scaled cluster abundance
Condition
infected child
infected adult
uninfected child
uninfected adult
BH-corrected p-values from KW and Wilcoxon testing
Adjusted p values
Comparisons
C
Intra-family comparisons
GCB-like 11
IgD+CD21+CXCR5high
DN 6
IgD-CD27-IgM+CD69+CD11c+
Relative abundance [%]
infected child
infected adult
D
Average PCA coordinates per patient
PC2 (9.04%)
PC1 (13.22%)
E
Mean scaled marker expression per patient
R = -0.41, p = 4.2e-07
CD21 scaled expression
Patient age
F
Monocytes and DCs
CD4+ T Cells
B Cells
Marker
Scaled mean expression
Group
infected child
infected adult

Figure S3. Annotation and statistical analysis of B-cell clusters identified by CyTOF, common cross-leukocyte pattern of age-dependency in infection-induced activation marker expression, related to Figure 2

(A) Heatmap, containing z-score normalized average expression values of each B-cell cluster resulting from a semi-supervised FlowSOM clustering approach of CyTOF data from whole blood samples (all the patients included in the dataset and used for clustering: uninfected children (RECAST n = 13; median age = 7), uninfected adults (RECAST n = 25; median age = 48), infected children (RECAST study n = 48; median age = 8; asymptomatic n = 11, symptomatic n = 37) and adults (RECAST n = 21, PA-COVID study n = 30; median age = 47; mild n = 37, severe n = 10, severe with IFN autoantibodies n = 4)). The dendrogram in the upper part of the panel shows the result of distance-based hierarchical clustering. A color-coded annotation of each cluster is presented on the right.

(B) Heatmap, showing scaled mean cluster abundance of B-cell clusters for each patient group (above) and a heatmap presenting the results of statistical testing for all relevant comparisons (below; Benjamini-Hochberg-corrected p values from non-parametric Kruskal-Wallis with post-hoc Wilcoxon). Severe patients with IFN autoantibodies were excluded from the analysis.

(C) Box plots, showing relative frequencies of annotated clusters, resulting from the FlowSOM algorithm, applied to the B-cell CyTOF data. Cluster abundance is compared between infected children (RECAST study n = 16; median age = 9; asymptomatic n = 2, symptomatic n = 14) and infected adults (RECAST study n = 14; median age = 38; mild n = 14) within the same family. Abundances were averaged, if multiple parents (mother and father) or children were measured in a family (family n = 11). Lines connect parents and children from the same family. Statistical testing was done using pairwise Wilcoxon rank sum test.

(D) PCA plot, presenting average principal component 1 and 2 coordinates of all B cells of each patient (colored according to patient group). The plot contains arrows labeled with marker names. These arrows represent the importance of each marker for the calculation of the components. The direction shows which principal components the marker correlates with and the length of the arrow illustrates the magnitude of the variable's contribution to the principal components. Simply put, each arrow points in the direction where the variable it represents increases in the principal component space and longer arrows indicate that the variable has a strong influence on the principal components. Severe patients with IFN autoantibodies were excluded from the analysis.

(E) Scatter plot, showing mean z-score normalized CD21 expression in relationship to patient age for infected patients (linear model fitted to data and Spearman's rank correlation coefficient shown in black) within B-cell CyTOF data. Severe patients with IFN autoantibodies were excluded from the analysis.

(F) Box plots, showing mean z-score normalized expression per patient for a curated list of markers for monocytes and DCs, $CD4^+$ T cells and B cells. Signals are compared between infected children (RECAST study n = 48; median age = 8; asymptomatic n = 11, symptomatic n = 37) and adults (RECAST n = 21, PA-COVID study n = 26; median age = 45; mild n = 37, severe n = 10). Wilcoxon; *, $p < 0.05$; **, $p < 0.01$; ***, $p < 0.001$; ****, $p < 0.0001$.

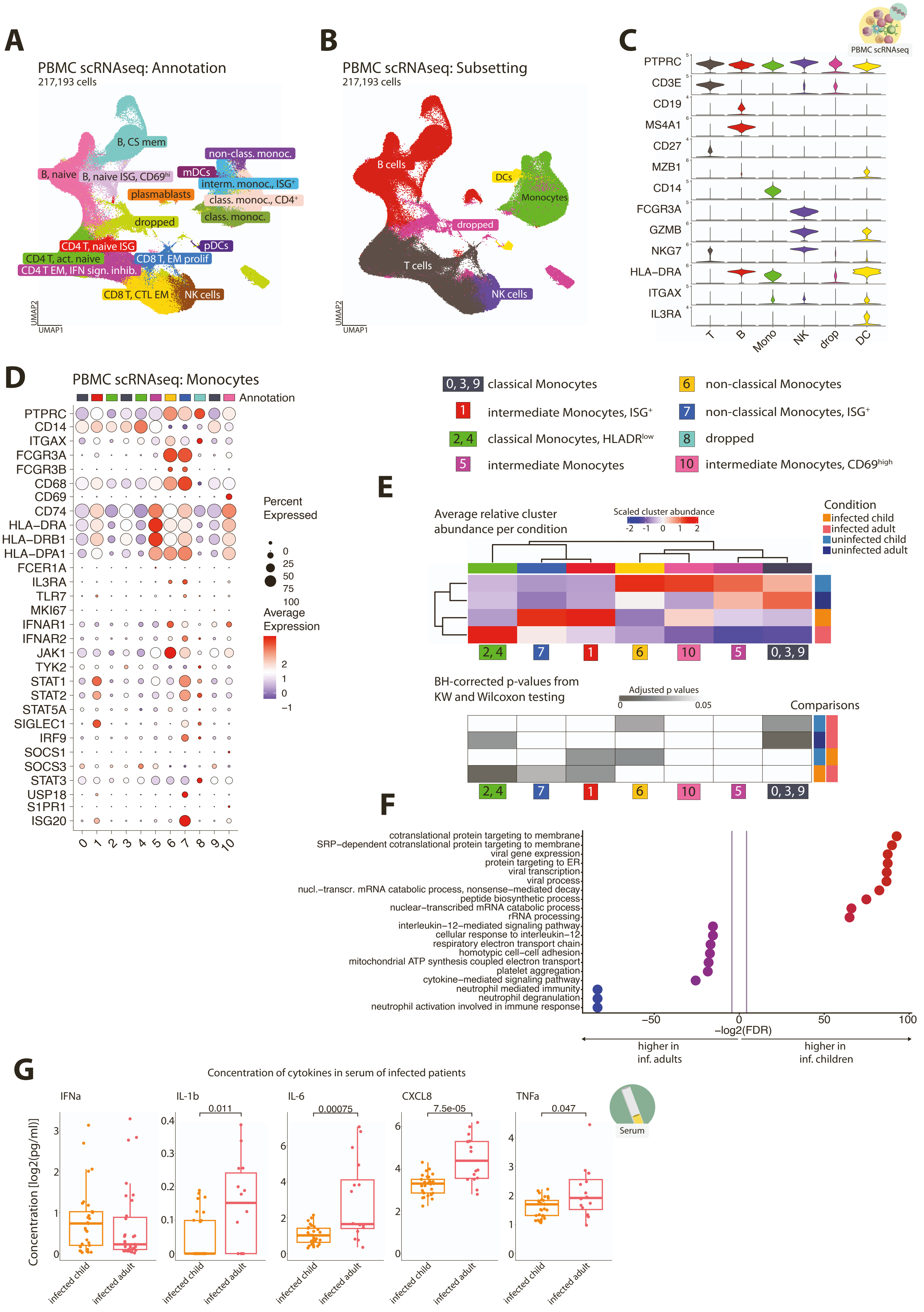
A
PBMC scRNAseq: Annotation
217,193 cells
B, CS mem
non-class. monoc.
B, naive
B, naive ISG, CD69hi
mDCs
interm. monoc., ISG+
plasmablasts
class. monoc., CD4+
class. monoc.
dropped
CD4 T, naive ISG
pDCs
CD4 T, act. naive
CD8 T, EM prolif
CD4 T EM, IFN sign. inhib.
CD8 T, CTL EM
NK cells
UMAP2
UMAP1
B
PBMC scRNAseq: Subsetting
217,193 cells
B cells
DCs
Monocytes
dropped
T cells
NK cells
UMAP2
UMAP1
C
PBMC scRNAseq
PTPRC
CD3E
CD19
MS4A1
CD27
MZB1
CD14
FCGR3A
GZMB
NKG7
HLA−DRA
ITGAX
IL3RA
T
B
Mono
NK
drop
DC
D
PBMC scRNAseq: Monocytes
Annotation
PTPRC
CD14
ITGAX
FCGR3A
FCGR3B
CD68
CD69
CD74
HLA−DRA
HLA−DRB1
HLA−DPA1
FCER1A
IL3RA
TLR7
MKI67
IFNAR1
IFNAR2
JAK1
TYK2
STAT1
STAT2
STAT5A
SIGLEC1
IRF9
SOCS1
SOCS3
STAT3
USP18
S1PR1
ISG20
0 1 2 3 4 5 6 7 8 9 10
Percent Expressed
0
25
50
75
100
Average Expression
2
1
0
−1
0, 3, 9 classical Monocytes
1 intermediate Monocytes, ISG+
2, 4 classical Monocytes, HLADRlow
5 intermediate Monocytes
6 non-classical Monocytes
7 non-classical Monocytes, ISG+
8 dropped
10 intermediate Monocytes, CD69high
E
Average relative cluster abundance per condition
Scaled cluster abundance
-2 -1 0 1 2
Condition
infected child
infected adult
uninfected child
uninfected adult
2, 4
7
1
6
10
5
0, 3, 9
BH-corrected p-values from KW and Wilcoxon testing
Adjusted p values
0
0.05
Comparisons
2, 4
7
1
6
10
5
0, 3, 9
F
cotranslational protein targeting to membrane
SRP−dependent cotranslational protein targeting to membrane
viral gene expression
protein targeting to ER
viral transcription
viral process
nucl.−transcr. mRNA catabolic process, nonsense−mediated decay
peptide biosynthetic process
nuclear−transcribed mRNA catabolic process
rRNA processing
interleukin−12−mediated signaling pathway
cellular response to interleukin−12
respiratory electron transport chain
homotypic cell−cell adhesion
mitochondrial ATP synthesis coupled electron transport
platelet aggregation
cytokine−mediated signaling pathway
neutrophil mediated immunity
neutrophil degranulation
neutrophil activation involved in immune response
−50
0
50
100
−log2(FDR)
higher in inf. adults
higher in inf. children
G
Concentration of cytokines in serum of infected patients
Serum
Concentration [log2(pg/ml)]
IFNa
IL-1b
0.011
IL-6
0.00075
CXCL8
7.5e-05
TNFa
0.047
infected child
infected adult

Figure S4. scRNAseq-based molecular profiling of circulating monocytes and cytokine concentrations in serum of infected patients point at increased inflammatory state in infected adults, related to Figure 3

(A) UMAP representation of the total PBMC scRNAseq data. 26 clusters have been produced using a graph-based approach as implemented in Seurat package (KNN graph with Louvain community detection). Clusters were then annotated based on the expression of the most commonly used marker genes of immune metapopulations. UMAP presents all the patients included in the dataset and used for clustering: uninfected children (RECAST study n = 5; median age = 7), uninfected adults (RECAST study n = 4, EICOV/COVIMMUNIZE study n = 4; median age = 66), infected children (RECAST study n = 13; median age = 9; asymptomatic n = 5, symptomatic n = 8) and infected adults (RECAST study n = 1; PA-COVID study n = 18; median age = 67; mild n = 5, severe n = 7, severe with IFN autoantibodies n = 7).

(B) UMAP representation of the total PBMC scRNAseq data. 26 annotated clusters from S3A were assigned a metapopulation tag, based on the expression of the most commonly used marker genes. These tags were then used for splitting the dataset into DC, monocyte, NK, T and B-cell subsets, which were then separately subclustered to achieve a more granular resolution. Clusters that were either a mix of different immune cell lineages or did not have a high enough PTPRC gene expression were annotated as "dropped" and excluded from further analysis.

(C) Violin plots, showing some of the marker genes used for the annotation of the metapopulations.

(D) Dotplot, showing average scaled expression of a curated, subset defining list of genes for monocyte cells, subset from the PBMC scRNAseq data. 11 clusters have been produced using a graph-based approach as implemented in Seurat package (KNN graph with Louvain community detection). Some clusters share annotation due to phenotypical similarity and accumulation pattern and are treated as one population for abundance testing in S3E. Annotations of the clusters are shown on the right-hand side from the dotplot. The cluster annotated as "dropped" was of low quality (as concluded from inspecting number of features, n of counts and percentage of mitochondrial genes as well as other population-specific genes). The dotplot presents all the patients included in the dataset and used for clustering: uninfected children (RECAST study n = 5; median age = 7), uninfected adults (RECAST study n = 4, EICOV/COVIMMUNIZE study n = 4; median age = 66), infected children (RECAST study n = 13; median age = 9; asymptomatic n = 5, symptomatic n = 8) and infected adults (RECAST study n = 1; PA-COVID study n = 18; median age = 67; mild n = 5, severe n = 7, severe with IFN autoantibodies n = 7).

(E) Heatmap, showing scaled mean cluster abundance of monocyte clusters for each patient group (above) and a heatmap presenting the results of statistical testing for all relevant comparisons (below; Benjamini-Hochberg-corrected p values from non-parametric Kruskal-Wallis with post-hoc Wilcoxon). Severe patients with IFN autoantibodies were excluded from the analysis.

(F) Gene set enrichment analysis (GSEA) applied to monocytes from the PBMC scRNAseq experiment and clusters, expanded with infection (1, 4 and 7). GSEA was done using the 189 significantly differentially expressed genes from the infected adults – infected children comparison, shown in volcano plot F3D. R interface EnrichR was used to access Enrichr "GO Biological Process 2018" database for automatic annotation of enriched gene sets. Severe patients with IFN autoantibodies were excluded from the analysis.

(G) Box plots of selected pro-inflammatory cytokines' serum concentrations in infected patients (inf. children RECAST study n = 29; median age = 9; asymptomatic n = 9, symptomatic n = 20; inf. adults RECAST study n = 5, PA-COVID study n = 26; median age = 54; mild n = 12, severe n = 14). Median DPSO in infected children = 5.5, median DPSO in infected adults = 9. Wilcoxon p values shown.

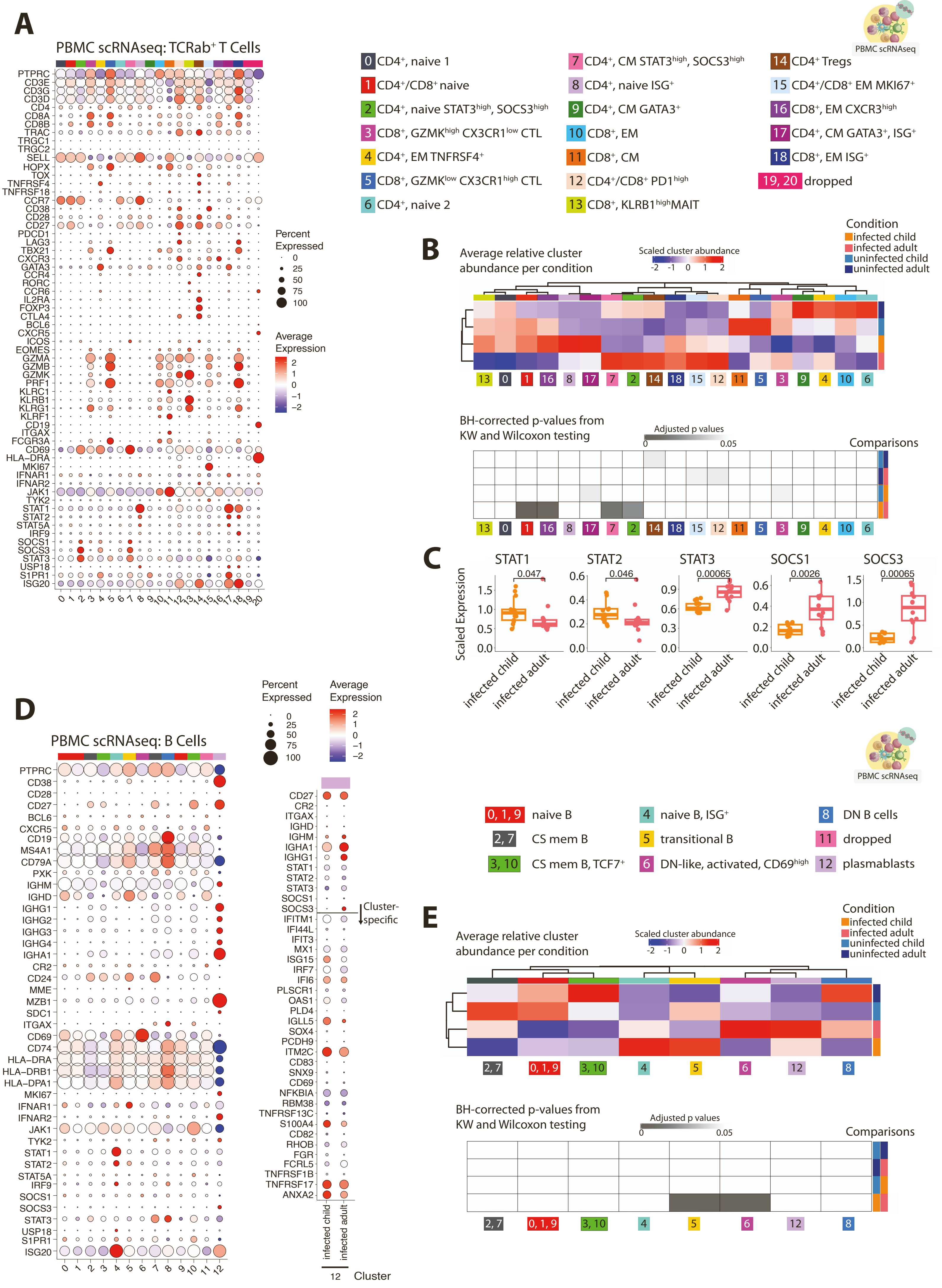
A
PBMC scRNAseq: TCRab+ T Cells
PTPRC CD3E CD3G CD3D CD4 CD8A CD8B TRAC TRGC1 TRGC2 SELL HOPX TOX TNFRSF4 TNFRSF18 CCR7 CD38 CD28 CD27 PDCD1 LAG3 TBX21 CXCR3 GATA3 CCR4 RORC CCR6 IL2RA FOXP3 CTLA4 BCL6 CXCR5 ICOS EOMES GZMA GZMB GZMK PRF1 KLRC1 KLRB1 KLRG1 KLRF1 CD19 ITGAX FCGR3A CD69 HLA-DRA MKI67 IFNAR1 IFNAR2 JAK1 TYK2 STAT1 STAT2 STAT5A IRF9 SOCS1 SOCS3 STAT3 USP18 S1PR1 ISG20
0 1 2 3 4 5 6 7 8 9 10 11 12 13 14 15 16 17 18 19 20
Percent Expressed
0
25
50
75
100
Average Expression
2
1
0
−1
−2
PBMC scRNAseq
0 CD4+, naive 1
1 CD4+/CD8+ naive
2 CD4+, naive STAT3high, SOCS3high
3 CD8+, GZMKhigh CX3CR1low CTL
4 CD4+, EM TNFRSF4+
5 CD8+, GZMKlow CX3CR1high CTL
6 CD4+, naive 2
7 CD4+, CM STAT3high, SOCS3high
8 CD4+, naive ISG+
9 CD4+, CM GATA3+
10 CD8+, EM
11 CD8+, CM
12 CD4+/CD8+ PD1high
13 CD8+, KLRB1highMAIT
14 CD4+ Tregs
15 CD4+/CD8+ EM MKI67+
16 CD8+, EM CXCR3high
17 CD4+, CM GATA3+, ISG+
18 CD8+, EM ISG+
19, 20 dropped
B
Average relative cluster abundance per condition
Scaled cluster abundance
-2 -1 0 1 2
Condition
infected child
infected adult
uninfected child
uninfected adult
13 0 1 16 8 17 7 2 14 18 15 12 11 5 3 9 4 10 6
BH-corrected p-values from KW and Wilcoxon testing
Adjusted p values
0 0.05
Comparisons
13 0 1 16 8 17 7 2 14 18 15 12 11 5 3 9 4 10 6
C
STAT1 STAT2 STAT3 SOCS1 SOCS3
Scaled Expression
0.047 0.046 0.00065 0.0026 0.00065
infected child infected adult
D
PBMC scRNAseq: B Cells
PTPRC CD38 CD28 CD27 BCL6 CXCR5 CD19 MS4A1 CD79A PXK IGHM IGHD IGHG1 IGHG2 IGHG3 IGHG4 IGHA1 CR2 CD24 MME MZB1 SDC1 ITGAX CD69 CD74 HLA-DRA HLA-DRB1 HLA-DPA1 MKI67 IFNAR1 IFNAR2 JAK1 TYK2 STAT1 STAT2 STAT5A IRF9 SOCS1 SOCS3 STAT3 USP18 S1PR1 ISG20
0 1 2 3 4 5 6 7 8 9 10 11 12
Percent Expressed
0
25
50
75
100
Average Expression
2
1
0
−1
−2
CD27 CR2 ITGAX IGHD IGHM IGHA1 IGHG1 STAT1 STAT2 STAT3 SOCS1 SOCS3 IFITM1 IFI44L IFIT3 MX1 ISG15 IRF7 IFI6 PLSCR1 OAS1 PLD4 IGLL5 SOX4 PCDH9 ITM2C CD83 SNX9 CD69 NFKBIA RBM38 TNFRSF13C S100A4 CD82 RHOB FGR FCRL5 TNFRSF1B TNFRSF17 ANXA2
Cluster-specific
infected child infected adult
12 Cluster
PBMC scRNAseq
0, 1, 9 naive B
2, 7 CS mem B
3, 10 CS mem B, TCF7+
4 naive B, ISG+
5 transitional B
6 DN-like, activated, CD69high
8 DN B cells
11 dropped
12 plasmablasts
E
Average relative cluster abundance per condition
Scaled cluster abundance
-2 -1 0 1 2
Condition
infected child
infected adult
uninfected child
uninfected adult
2, 7 0, 1, 9 3, 10 4 5 6 12 8
BH-corrected p-values from KW and Wilcoxon testing
Adjusted p values
0 0.05
Comparisons
2, 7 0, 1, 9 3, 10 4 5 6 12 8

Figure S5. scRNAseq profiling of TCRab$^+$ T-cell and B-cell space, related to Figure 4

(A) Dotplot, showing average scaled expression of a curated, subset defining list of genes for TCRab$^+$ T cells, subset from the PBMC scRNAseq data. 21 clusters have been produced using a graph-based approach as implemented in Seurat package (KNN graph with Louvain community detection). Annotations of the clusters are shown on the right-hand side from the dotplot. Clusters annotated as "dropped" were of low quality (as concluded from inspecting number of features, n of counts and percentage of mitochondrial genes as well as other population-specific genes). The dotplot presents all the patients included in the dataset and used for clustering: uninfected children (RECAST study n = 5; median age = 7), uninfected adults (RECAST study n = 4, EICOV/COVIMMUNIZE study n = 4; median age = 66), infected children (RECAST study n = 13; median age = 9; asymptomatic n = 5, symptomatic n = 8) and infected adults (RECAST study n = 1; PA-COVID study n = 18; median age = 67; mild n = 5, severe n = 7, severe with IFN autoantibodies n = 7).

(B) Heatmap, showing scaled mean cluster abundance of TCRab$^+$ T-cell clusters for each patient group (above) and a heatmap presenting the results of statistical testing for all relevant comparisons (below; Benjamini-Hochberg-corrected p values from non-parametric Kruskal-Wallis with post-hoc Wilcoxon). Severe patients with IFN autoantibodies were excluded from the analysis.

(C) Box plots, showing mean scaled expression of selected genes, involved in IFN signaling, calculated over all TCRab$^+$ T cells from clusters expanded with infection (2, 7, 8, 12, 15, 16, 17 and 18) for infected children (RECAST study n = 13; median age = 9; asymptomatic n = 5, symptomatic n = 8) and infected adults (RECAST study n = 1; PA-COVID study n = 11; median age = 63.5; mild n = 5, severe n = 7). Wilcoxon p values shown.

(D) Left hand side: dotplot, showing average scaled expression of a curated, subset defining list of genes for B cells, subset from the PBMC scRNAseq data. 13 clusters have been produced using a graph-based approach as implemented in Seurat package (KNN graph with Louvain community detection). Annotations of the clusters are shown on the right-hand side from the dotplot. Cluster annotated as "dropped" was of low quality (as concluded from inspecting number of features, n of counts and percentage of mitochondrial genes as well as other population-specific genes). The dotplot presents all the patients included in the dataset and used for clustering: uninfected children (RECAST study n = 5; median age = 7), uninfected adults (RECAST study n = 4, EICOV/COVIMMUNIZE study n = 4; median age = 66), infected children (RECAST study n = 13; median age = 9; asymptomatic n = 5, symptomatic n = 8) and infected adults (RECAST study n = 1; PA-COVID study n = 18; median age = 67; mild n = 5, severe n = 7, severe with IFN autoantibodies n = 7). Right hand side: Dotplot, showing scaled average expression of genes in cluster 12, split by infected patient group. Severe patients with IFN autoantibodies were excluded from the analysis. A horizontal line splits the dotplot in two parts – genes above the line were curated based on the presence of clusters with pronounced ISG signature and include other genes useful for annotation, genes below the line were found to be differentially expressed between the clusters (FindMarkers Seurat function).

(E) Heatmap, showing scaled mean cluster abundance of B-cell clusters for each patient group (above) and a heatmap presenting the results of statistical testing for all relevant comparisons (below; Benjamini-Hochberg-corrected p values from non-parametric Kruskal-Wallis with post-hoc Wilcoxon). Severe patients with IFN autoantibodies were excluded from the analysis.

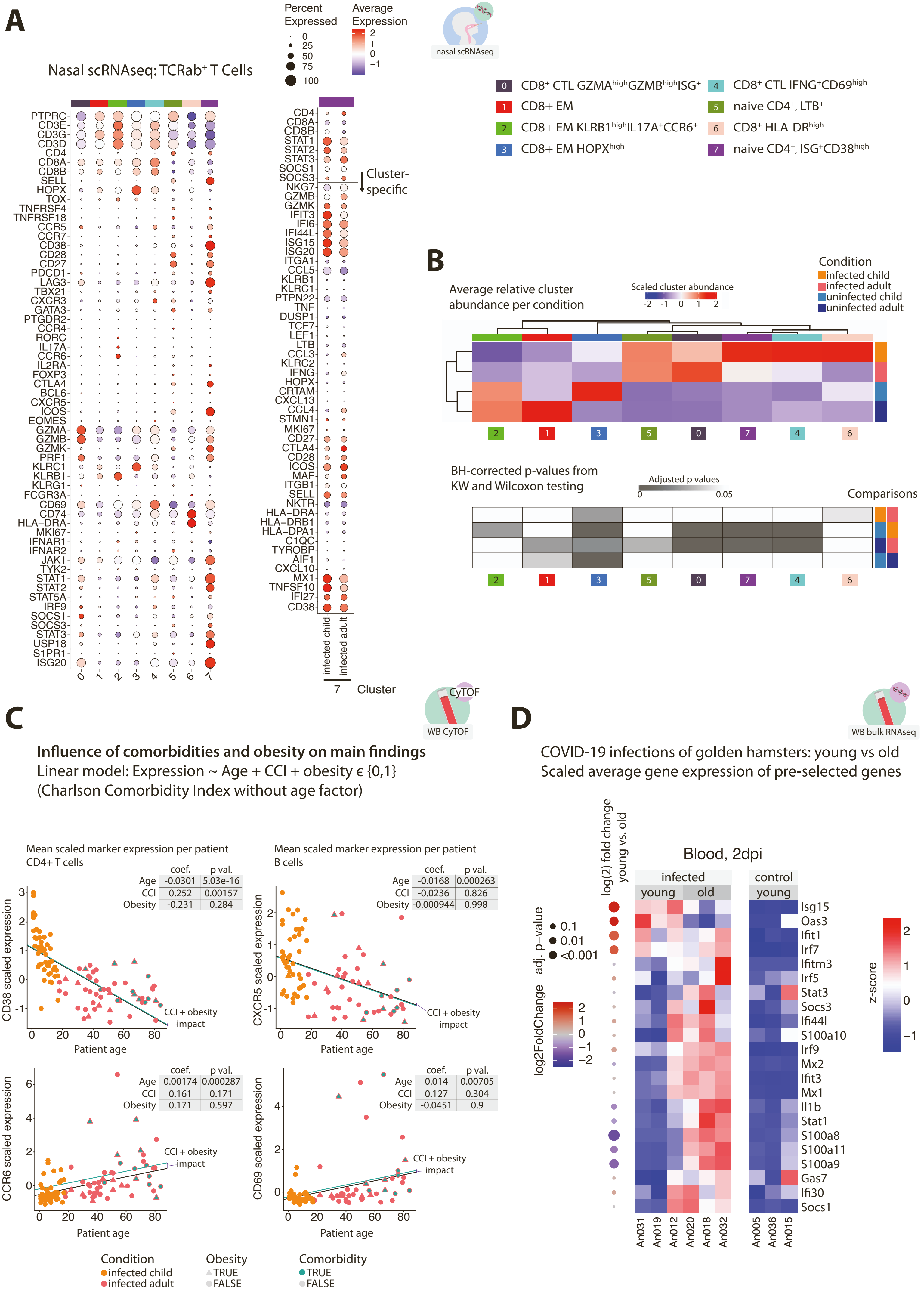

	coef.	p val.
Age	-0.0301	5.03e-16
CCI	0.252	0.00157
Obesity	-0.231	0.284

	coef.	p val.
Age	-0.0168	0.000263
CCI	-0.0236	0.826
Obesity	-0.000944	0.998

	coef.	p val.
Age	0.00174	0.000287
CCI	0.161	0.171
Obesity	0.171	0.597

	coef.	p val.
Age	0.014	0.00705
CCI	0.127	0.304
Obesity	-0.0451	0.9

Figure S6. Age-dependent activation profiles are not driven by comorbidities and can be confirmed in a hamster infection model, related to Figure 5

(A) Left hand side: dotplot, showing TCRab$^+$ cells, subset from the nasal swab scRNAseq data.[8] 8 clusters have been produced using a graph-based approach as implemented in Seurat package (KNN graph with Louvain community detection). Annotations of the clusters are shown on the right-hand side from the dotplot. The dotplot presents all the patients included in the dataset and used for clustering: uninfected children (RECAST study n = 18; median age = 9), uninfected adults (RECAST study n = 23; median age = 46), infected children (RECAST study n = 24; median age = 9; asymptomatic n = 2, symptomatic n = 22) and infected adults (RECAST study n = 21; median age = 39; mild n = 21). Patients with DPSO range from 0 to 12 days were included (inf. children mean = 4.5, inf. adult mean = 5). Right hand side: dotplot, showing scaled average expression of genes in cluster 7, split by infected patient group. Severe patients with IFN autoantibodies were excluded from the analysis. A horizontal line splits the dotplot in two parts – genes above the line were curated based on the presence of clusters with pronounced ISG signature and include other genes useful for annotation, genes below the line were found to be differentially expressed between the clusters (FindMarkers Seurat function).

(B) Heatmap, showing scaled mean cluster abundance of TCRab$^+$ cell clusters for each patient group (above) and a heatmap presenting the results of statistical testing for all relevant comparisons (below; Benjamini-Hochberg-corrected p values from non-parametric Kruskal-Wallis with post-hoc Wilcoxon).

(C) Scatterplots, illustrating the results of modelling dependency between age and mean z-score normalized CD38 and CCR6 expression by CD4$^+$ T cells or CXCR5 and CD69 expression by B cells from the CyTOF experiment together with Charlson Comorbidity Index (CCI, w/o age factor) and obesity (as binary variable, 1 if BMI > 30). Light green line shows the impact of CCI and obesity on the intercept value of the model (intercept + CCI coefficient + obesity coefficient). CCI score was calculated excluding age, as age is already present in the model as a separate variable. Distance between the two lines thus represents the influence of the CCI score and obesity on the intercept (mean value of the response variable when all the predictor variables are zero). Obesity status is marked by triangular shape of a point, comorbidity by a light green fill. Figure annotation includes the respective coefficients and p values as modelled with linear regression. Infected children (RECAST study n = 48; median age = 8; asymptomatic n = 11, symptomatic n = 37) and adults (RECAST n = 21, PA-COVID study n = 26; median age = 45; mild n = 37, severe n = 10) are shown.

(D) Heatmaps, summarizing the differential gene expression analysis results of gene sets identified to be up-regulated in infection-induced leukocyte populations (in human PBMC scRNAseq data) determined by bulk RNA sequencing of whole blood samples collected at day 2 after infection from young (n=3, 6 weeks old) and old (n=3, 32-34 weeks old) hamsters (left side) as well as uninfected controls (n=3, right side, 6 weeks old).

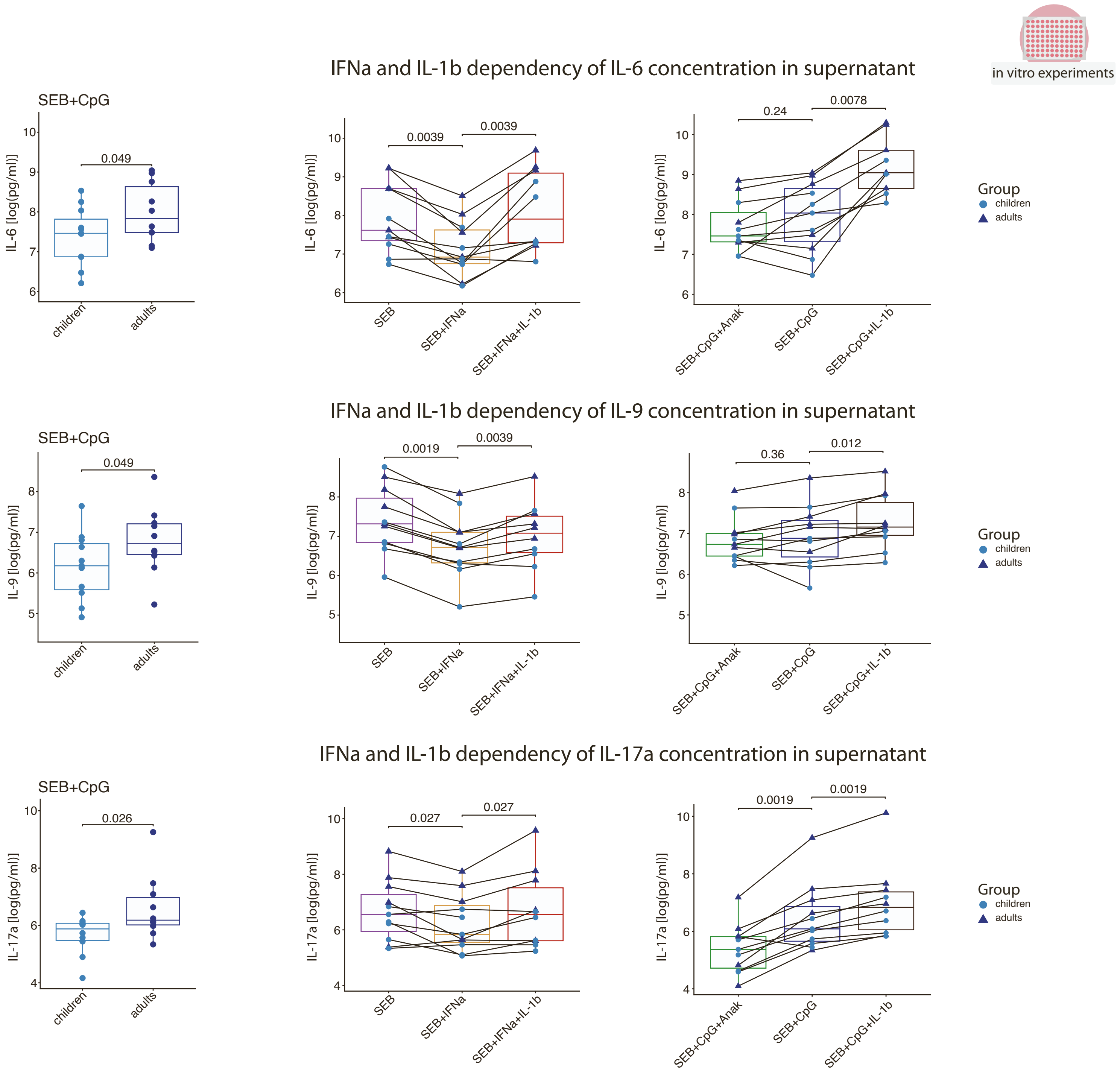

in vitro experiments
IFNa and IL-1b dependency of IL-6 concentration in supernatant
SEB+CpG
IL-6 [log(pg/ml)]
0.049
children
adults
0.0039
0.0039
SEB
SEB+IFNa
SEB+IFNa+IL-1b
0.24
0.0078
SEB+CpG+Anak
SEB+CpG
SEB+CpG+IL-1b
Group
children
adults
IFNa and IL-1b dependency of IL-9 concentration in supernatant
SEB+CpG
IL-9 [log(pg/ml)]
0.049
0.0019
0.0039
0.36
0.012
IFNa and IL-1b dependency of IL-17a concentration in supernatant
SEB+CpG
IL-17a [log(pg/ml)]
0.026
0.027
0.027
0.0019
0.0019

Figure S7. IL-1b-mediated interference with type I IFN signaling explains enhanced cytokine production potential of adult leukocytes, related to Figure 6

Box plots showing the difference in IL-6, IL-9 and IL-17A release between the two age groups when stimulated by SEB and CpG (first column from the left; uninfected children, RECAST study n = 11; median age = 9; uninfected adult, RECAST study n = 10; median age = 55; one-sided Wilcoxon p value shown), or by different combinations of SEB, CpG, recombinant IFNa (0.5ng/ml), IL-1b and IL-1b inhibitor Anakinra (uninfected children, RECAST study n = 6; median age = 8; uninfected adults, RECAST study n = 5; median age = 55; p values shown for paired Wilcoxon test, adjusted with Hommel procedure).

Table S2: Cohort summary and statistics, related to all Figures.				
	**Uninfected children**	**Uninfected adults**	**Infected children**	**Infected adults**
Total number	22	49	58	98
- RECAST	22	35	58	40
- PA-COVID	0	0	0	58
- COVIMMUNIZE, EICOV	0	14	0	0
Age, median (range)	8.5 (2–16)	52 (25-86)	8 (1-17)	47 (18-84)
Sex				
- Male, N (%)	10 (45%)	24 (49%)	30 (52%)	51 (52%)
- Female, N (%)	12 (55%)	25 (51%)	28 (48%)	47 (48%)
COVID-19, WHO scale, N (%)				
- Asymptomatic (1)	0 (0%)	0 (0%)	11 (19%)	0 (0%)
- Mild (2-4)	0 (0%)	0 (0%)	47 (81%)	68 (69%)
- Severe (5-8)	0 (0%)	0 (0%)	0 (0%)	30 (31%)
DPSO first visit, median±SD, mean (range)	n/a	n/a	4.5±3.3<br>5<br>(-1-11)	8±4.5<br>7.7<br>(-3-19)
DPSO second visit, median±SD, mean (range)	n/a	n/a	20±3.8<br>20<br>(12-30)	22±5.3<br>22<br>(12-41)
DPSO third visit, median±SD, mean (range)	n/a	n/a	147±16.2<br>153<br>(129-183)	181±26.4<br>176<br>(122-245)
CCI w/o age factor > 0, N (%), median CCI w/o age factor (range)	0 (0%)<br>0 (n/a)	9 (18.4%)<br>0 (0-4)	2 (3.5%)<br>0 (0-1)	30 (30.6%)<br>0 (0-7)
- Myocardial infarction, N (%)	0 (0%)	0 (0%)	0 (0%)	3 (3.1%)
- Chronic heart failure, N (%)	0 (0%)	0 (0%)	0 (0%)	0 (0%)
- Peripheral artery disease, N (%)	0 (0%)	1 (2%)	0 (0%)	2 (2%)
- Cerebrovascular accident / transient ischemic stroke, N (%)	0 (0%)	2 (4.1%)	0 (0%)	1 (1%)
- Dementia, N (%)	0 (0%)	1 (2%)	0 (0%)	0 (0%)
- Chronic obstructive pulmonary disease, N (%)	0 (0%)	2 (4.1%)	0 (0%)	7 (7.1%)
- Connective tissue disorder, N (%)	0 (0%)	2 (4.1%)	0 (0%)	2 (2%)
- Peptic ulcer disease, N (%)	0 (0%)	0 (0%)	0 (0%)	0 (0%)
- Chronic liver disease, N (%)	0 (0%)	0 (0%)	0 (0%)	4 (4.1%)
- Diabetes, N (%)	0 (0%)	0 (0%)	2 (3.5%)	17 (17.3%)
- Chronic kidney disease, N (%)	0 (0%)	0 (0%)	0 (0%)	6 (6.1%)
- Tumor, N (%)	0 (0%)	5 (10.2%)	0 (0%)	3 (3.1%)
- Leukaemia, N (%)	0 (0%)	0 (0%)	0 (0%)	0 (0%)
- Lymphoma, N (%)	0 (0%)	0 (0%)	0 (0%)	0 (0%)
- AIDS, N (%)	0 (0%)	0 (0%)	0 (0%)	1 (1%)
Other COVID-relevant comorbidities				
- Asthma, N (%)	1 (4.5%)	3 (6.1%)	1 (1.7%)	5 (5.1%)
- Obesity, N (%)<br>*only 40 uninf. adult patients with BMI available	1 (4.5%)	2 (4.1%)*	1 (1.7%)	23 (23.5%)

Table S3: Patient kinship annotation for intra-family comparisons, related to Figures S2D, S3C.

patient_id	family_id	category
468	1	adult
471	1	child
481	2	adult
482	2	adult
483	2	child
50001	3	child
50004	3	adult
50005	4	child
50006	4	child
50007	4	adult
50017	5	adult
50019	5	child
50026	6	child
50050	7	adult
50051	7	child
50053	7	adult
50057	8	child
50058	8	adult
50108	9	child
50110	9	adult
50126	10	child
50128	10	adult
50129	10	adult
50134	11	adult
50135	11	adult
50136	11	child
50137	11	child
50138	12	adult
50139	12	adult
50140	12	child
50143	13	adult
50144	13	child
50165	14	adult
50166	14	child
50170	15	child
50171	15	child
50172	15	child
50174	15	adult
50181	16	adult
50182	16	child
50183	16	child
